# Structures of the *P. aeruginosa* FleQ-FleN master regulators reveal large-scale conformational switching in motility and biofilm control

**DOI:** 10.1073/pnas.2312276120

**Published:** 2023-12-05

**Authors:** Lucía Torres-Sánchez, Thibault Géry Sana, Marion Decossas, Yaser Hashem, Petya Violinova Krasteva

**Affiliations:** ^a^Université de Bordeaux, CNRS, Bordeaux INP, CBMN, UMR 5248, Pessac F-33600, France; ^b^Structural Biology of Biofilms Group, European Institute of Chemistry and Biology, Pessac F-33600, France; ^c^Doctoral School of Therapeutic Innovation (ITFA), Université Paris-Saclay, Gif-sur-Yvette F-91190, France; ^d^ARNA Laboratory, European Institute of Chemistry and Biology, U1212 INSERM, UMR5320 CNRS, Université de Bordeaux, Pessac F-33600, France

**Keywords:** bacterial biofilms, cyclic dinucleotides, c-di-GMP, Cryo-EM, gene regulation

## Abstract

*Pseudomonas aeruginosa* can cause a wide array of chronic and acute infections associated with its ability to rapidly switch between planktonic, biofilm, and dispersed lifestyles, each with a specific arsenal for bacterial survival and virulence. At the cellular level, many of the physiological transitions are orchestrated by the intracellular second messenger c-di-GMP and its receptor-effector FleQ. A bacterial enhancer binding protein, FleQ acts as a master regulator of both flagellar motility and adherence factor secretion and uses remarkably different transcription activation mechanisms depending on its dinucleotide loading state, adenosine triphosphatase (ATPase) activity, interactions with polymerase sigma (σ) factors, and complexation with a second ATPase, FleN. How the FleQ-FleN tandem can exert diverse effects through recognition of a conserved FleQ binding consensus has remained enigmatic. Here, we provide cryogenic electron microscopy (cryo-EM) structures of both c-di-GMP-bound and c-di-GMP-free FleQ-FleN complexes which deepen our understanding of the proteins’ (di)nucleotide-dependent conformational switching and fine-tuned roles in gene expression regulation.

## Significance

*Pseudomonas aeruginosa* is an ESKAPE pathogen that causes a wide array of chronic and acute infections due to its ability to rapidly switch between planktonic, biofilm-forming, and dispersed lifestyles. FleQ and FleN act as master regulators of both flagellar motility and biofilm formation, while using different transcription activation mechanisms dependent on the target promoter and the presence or absence of the second messenger cyclic diguanylate (c-di-GMP). Here, we present c-di-GMP-bound and dinucleotide-free cryogenic electron microscopy structures of FleQ-FleN complexes and provide an updated functional model for gene expression regulation at flagellar and biofilm-related promoters. Our data not only present near-atomic resolution blueprints of these key molecular switches but also redress and integrate fragmentary biochemical, functional, and structural data previously reported.

*Pseudomonas aeruginosa* is a widespread Gram-negative bacterium and a highly adaptable opportunistic pathogen of increasing medical importance. It affects primarily immunocompromised individuals, such as cystic fibrosis patients, implant recipients and burn victims, and can cause not only severe chronic infections, but also acute pneumonias and septic shock conditions (1). The pathogen’s remarkable versatility in infection strategies is by and large dependent on its ability to switch from a motile lifestyle during host invasion; through extracellular matrix secretion, biofilm formation, and immune escape; to biofilm dispersal and acute state virulence (2, 3). Most of these transitions are associated with pathway-specific signaling events dependent on the second messenger cyclic diguanylate (c-di-GMP), whose elevated levels are generally associated with the inhibition of flagellar motility and the induced secretion of adherence factors such as exopolysaccharides and cell surface adhesins (4, 5). C-di-GMP-dependent regulation can occur at multiple levels and through a diverse range of intracellular targets, of which the FleQ transcription regulator represents an impressively versatile sensor-effector (5).

**Fig. 1.**
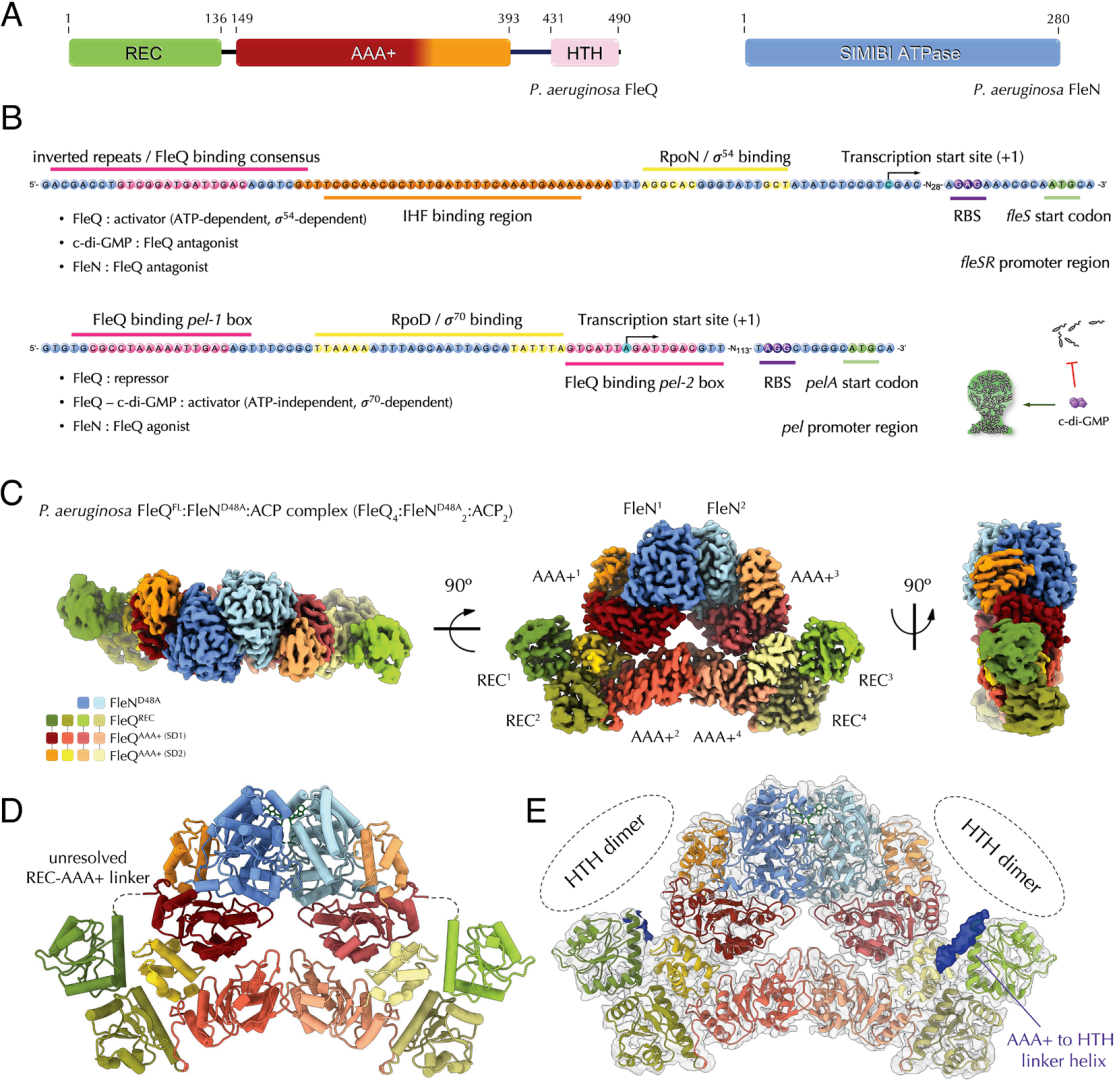
The FleQ and FleN regulators: functional roles and cryo-EM structure of the c-di-GMP-free complex. (*A*) Domain architectures of *P. aeruginosa* FleQ and FleN. (*B*) Example flagellar and biofilm-related promoters under FleQ control. Above, organization of the flagellar *fleSR* promoter subject to FleQ-, σ^54^-, and ATP-dependent regulation. RBS: ribosome-binding site; IHF: Integration Host Factor. Below, organization of the biofilm-promoting *pel* promoter region subject to σ^70^-dependent, ATP-independent regulation. Effects of c-di-GMP are summarized as inhibition of flagellar motility and induction of biofilm formation on the *Right*. (*C*) Segmented, color-coded electron density map of the purified FleQ^FL^-FleN^D48A^ complex bound to the nonhydrolysable ATP homolog AppCp (adenosine-5'-[(β,γ)-methyleno)triphosphate or ACP), as resolved by cryo-EM. (*D*) Cartoon representation of the solved FleQ_4_:FleN^D48A^_2_:ACP_2_ cryo-EM structure, color-coded as in *C*. (*E*) Map-to-model fit showing the putative formation of FleQ^HTH^ dimers (not resolved in the electron density map) as formed between adjacent FleQ protomers. Tubular densities likely corresponding to N-proximal helices from the AAA+-to-HTH domain linkers of the peripheral FleQ copies are shown in dark blue.

FleQ belongs to the NtrC subfamily of bacterial enhancer binding proteins (bEBP) and has conserved FleQ/FlrA/FlaK/FlgR homologs in *Vibrio cholerae* (6)*, Vibrio parahaemolyticus* (7)*, Vibrio alginolyticus* (8), *Shewanella putrefaciens* (9), and *Helicobacter pylori* (10), among others. The protein comprises an N-terminal receiver-like FleQ domain (FleQ^REC^) and a C-terminal helix-turn-helix DNA-binding motif (FleQ^HTH^). In between is a central bilobal AAA+ ATPase domain (FleQ^AAA+^) composed of an N-proximal α/β subdomain (SD1) and a C-proximal α-helical SD2 module (11) (Fig. 1*A*). A generally proposed activation mechanism for bEBPs of similar domain architecture is REC domain phosphorylation-driven switch from inactive dimers into adenosine triphosphate- (ATP-) and DNA-bound ring- or superhelical hexamers that recognize enhancer regulatory sequences typically upstream from the transcription start site of regulated promoters. ATP complexation occurs between adjacent “spooned” protomers, and upon interactions of the bEBP with promoter-bound, σ^54^–loaded RNA polymerase (RNAP), cooperative ATP hydrolysis would lead to remodeling of the closed RNAP-promoter complex (RPc) into the transcriptionally active state (12–14) (RPo, *SI Appendix*, Fig. S1*A*).

Mechanistically, FleQ and its close homologs differ substantially from this generic activation model. Significant sequence divergence from canonical response regulators renders the N-terminal FleQ^REC^ domain phosphorylation-incompetent, whereas dimerization of this N-terminal module is driven by an atypical α_1_ interface important for both motility and biofilm formation (15). Furthermore, the protein’s ATPase activity is constitutively active in solution and in the absence of DNA binding or RNAP subunits (16). Crystal structures of the apo-, ADP- and ATP-bound states of the central AAA+ domain show superhelical assemblies consistent with the typical spooned subunit contacts in active hexameric split-ring assemblies of bEBPs, and mutational analyses have confirmed the role of both *cis* and *trans* motifs in nucleotide coordination and hydrolysis (11, 14). FleQ exists primarily as dimers in solution with or without (di)nucleotide-based cofactors and exhibits cooperative ATPase activity with a Hill coefficient of ~2.5 (16). Together, these data suggest that a dominant spooned dimer conformation is likely the minimal requirement for enzymatic activity but higher-order cooperative oligomeric states are also sampled in solution. Indeed, such multimeric states, including hexamers, have been detected in solution in both *P. aeruginosa* FleQ and in its *V. alginolyticus* homolog FlaK (8, 11, 16) and could further influence FleQ ATPase activity and/or σ^54^–dependent RPc to RPo transition at flagellar promoters in vivo.

A combination of experimental and in silico studies have provided a comprehensive map of FleQ-binding sequences in the *P. aeruginosa* genome (17, 18), and in silico analysis of the putative DNA recognition mechanism (see *Results and Discussion* below) is consistent with a FleQ^HTH^ dimer binding to the identified pseudopalindromic DNA consensus (17). Consistent with its initially identified role as a master regulator of flagellar motility (19, 20), FleQ binds directly to the promoters of several flagellar gene operons, whose promoter architecture supports σ^54^- and ATP-dependent transcription activation (17, 18) (Fig. 1*B*). Among the induced genes is *fleN* (also known as *flhG*, *ylxH*, or *motR* in other species), encoding a weak ATPase from the ancient SIMIBI (Signal recognition particle, MinD, and BioD) superfamily of pro- and eukaryotic nucleoside triphosphatases (20–22). SIMIBI proteins are key to a large variety of cellular processes including divisome positioning, exopolysaccharide secretion system assembly, and membrane protein sorting (21, 23, 24). Importantly, SIMIBI function is almost invariably dependent on NTP-driven “sandwich” dimer formation, where nucleotide binding is uncoupled from hydrolysis and which secures a specific functional state in binding partner regulation (21, 23, 24). More than two decades ago, it was shown that disruption of the *fleN* gene leads to a multiflagellate phenotype and the upregulation of *fleQ*-dependent flagellar gene expression (25), and subsequent studies have confirmed that FleN directly binds FleQ to dampen its ATPase activity in vitro (16).

Recent crystallographic studies have proposed that this inhibition occurs in an ATP- and FleN dimerization-dependent manner and have shown that FleN can form a 2:2 complex with the isolated FleQ^AAA+^ domain (26) (*SI Appendix*, Fig. S1*B*). In it, a sandwich dimer of ATP-bound FleN is complexed on each side by a FleQ^AAA+^ protomer in a way that obstructs the latter’s ATP-binding active site and likely stabilizes the σ^54^-interacting G^221^AFTGA loop into an inactive conformation (26). The authors thus proposed a simple mechanism for flagellar motility regulation, where FleQ-induced FleN will cycle between an ATP-bound dimeric state to disrupt FleQ hexamer formation and DNA-binding, and a posthydrolysis state that would allow basal flagellar gene expression through hexamer reconstitution and σ^54^-dependent transcription activation (26) (*SI Appendix*, Fig. S1*B*).

Nevertheless, such a reductive model cannot reconcile important biochemical and genetic data acquired on both FleQ and FleN homologs. As mentioned above, FleQ exist primarily and likely binds DNA as a dimer, recognizing a conserved 14-nucleotide pseudopalindromic consensus (16, 17). Several studies have shown that FleQ binds DNA very weakly, independent of the presence of (di)nucleotides, and that FleN is actually necessary for efficient DNA complexation in the presence of either ATP or ADP as a cofactor (17, 27, 28). Whereas weak DNA binding in the absence of FleN could be possibly overcompensated by FleQ oligomerization and increased cooperativity between subunits, thus leading to hyperflagellation, in a wild-type context and consistent with the available biochemical data, nucleotide-bound FleN could actually free FleQ^HTH^ from an autoinhibited conformation to stimulate DNA consensus recognition. In such a scenario ATP-bound FleN would necessarily inhibit FleQ through a regulatory mechanism that is distinct from the FleN-mediated promoter release proposed recently (*SI Appendix*, Fig. S1*B*) (26).

Apart from flagellar gene promoters, FleQ was also found to recognize the promoters of multiple factors involved in biofilm formation, such as cell surface adhesins and exopolysaccharides (e.g., the *cdrAB*, *pel*, and *psl* clusters) (17, 18) (Fig. 1*B*). Interestingly, the majority of these promoters feature more than one FleQ-binding consensus and are actually inhibited by FleQ-FleN-ATP binding in the absence of c-di-GMP (17, 29). For at least some of them, such as the *pel* promoter which features an upstream FleQ-binding box 1 and a second one, box 2, overlapping with the transcription start site (Fig. 1*B*), it has been confirmed that the FleN-complexed FleQ binds to more than one site simultaneously, thus leading to altered promoter architecture via DNA bending and transcriptional repression (29). C-di-GMP complexation appears to relax the bent promoter architecture and liberates the transcription start site, whereas the box1-bound FleQ-FleN-c-di-GMP complex converts into a potent transcription activator (29). Strikingly, conserved -10 and -35 boxes in the promoter DNA indicate that transcription initiation is actually σ^70^- and not σ^54^-dependent (Fig. 1*B*). Finally, ATP hydrolysis is not required for either of the two ATPases and is actually inhibited upon c-di-GMP complexation (28, 29).

Whereas a c-di-GMP-bound FleQ^AAA+-HTH^ construct crystallized previously as a trimer of elongated dimers incompatible with ATP hydrolysis (11), the observed crystal contacts in that assembly are themselves at odds with the FleQ^AAA+^-FleN contacts reported recently (26). Therefore it remains unclear what the actual ATP- and c-di-GMP-dependent conformational states are within the FleQ-FleN complex and how that would affect transcription activation and/or repression on both σ^70^- and σ^54^-dependent promoters.

To gain further mechanistic insights and avoid crystallographic artifacts, we resorted to single-particle cryogenic electron microscopy (cryo-EM) on FleQ-FleN complexes and present here the solution structures of both the c-di-GMP-bound and c-di-GMP-free states. Both reveal largely preserved FleN-FleN and FleN-FleQ^AAA+^ contacts consistent with the recently reported crystal structure (26), confirming that the said assembly is not sufficient to explain FleQ’s functional and conformational plasticity. In addition, the cryo-EM structures provide insights into interactions of the essential regulatory FleQ^REC^ module with the protein’s catalytic core and corroborate roles of the atypical α_1_ dimerization interface (15) in both the dinucleotide-free and c-di-GMP-bound states. Surprisingly, however, the ATP homolog-bound FleQ-FleN complex features an unexpected 4:2 FleQ:FleN symmetric assembly, which supports an alternative model for FleN dimerization-dependent FleQ ATPase inhibition and provides insight into the nucleotide-dependent repression and DNA bending at biofilm-related promoters. In contrast, the c-di-GMP-bound state demonstrates an intrinsically asymmetric 3:2 FleQ:FleN architecture with striking reorientation of FleQ’s REC and AAA+ modules. This leads to a yet different mode of FleQ ATPase inhibition and unilateral complex disassembly at physiological (di)nucleotide concentrations, that could explain both repression of σ^54^-dependent flagellar gene expression, as well as DNA bending release and σ^70^-dependent expression activation at biofilm-promoting clusters.

## Results and Discussion

### Determinants for Stable FleQ-FleN Complex Formation In Cellulo.

As mentioned above, recent work demonstrated that the isolated FleQ^AAA+^ domain is necessary and sufficient for FleN complexation and that both proteins can crystallize in a 2:2 heterotetrameric assembly (26) (*SI Appendix*, Fig. S1*B*). Nevertheless, both solution binding and crystallization assays were performed at high protein concentrations, saturating concentrations of a nonhydrolysable ATP homolog, and non-natural diffusion-limiting factors, such as chemical modification and surface immobilization, or incubation with high-viscosity agents (26). To obtain a more physiological context on the structural determinants necessary for stable FleQ-FleN interaction, we resorted to in cellulo complex reconstitution using recombinant protein coexpression (*SI Appendix*, Fig. S2). We designed several FleQ constructs covering the conserved FleQ^REC^ domain (FleQ^1-139^), the FleQ^REC-AAA+^ tandem (FleQ^1-394^), the isolated FleQ^AAA+^ domain (FleQ^138-394^), the FleQ^AAA+-HTH^ tandem (FleQ^138-490^), a variant missing the last 13 amino acids based on previously published stable constructs (FleQ^FL*^ or FleQ^1-477^) (11), and the full-length protein (FleQ^FL^) and cloned them into coexpression-compatible vectors. Whereas N-terminally tagged FleQ failed to copurify stably untagged FleN, FleN used as bait was able to efficiently pull-down epitope-free FleQ variants only in constructs retaining both the receiver (REC) and central AAA+ ATPase modules (FleQ^FL^, FleQ^FL*^ and FleQ^REC-AAA+^) (*SI Appendix*, Fig. S2*B*). Conversely, neither FleQ^REC^ nor FleQ^AAA+^ alone were able to copurify stably with FleN. It is important to note that these experiments were performed in a minimal buffer at close to physiological pH and salt concentrations, and in the absence of any nucleotide-based cofactors, chemical modifications, or FleQ concentration during the hours-long purification procedure. Furthermore, following lysate incubation with the immobilized metal affinity chromatography (IMAC) resin, the latter was subject to stringent washes with large volumes of nucleotide-free buffer that could potentially release in cellulo–bound cofactors. Even though FleQ-FleN complexes purified via the ATP-binding but hydrolysis-incompetent FleN^D48A^ mutant were found to be more stable for structural studies than the ones using wild-type protein as bait, we reasoned that nucleotide-free, monomeric FleN can still bind FleQ in the cell.

Previous structural work on the isolated FleN protein has shown that in the presence of ATP it forms the typical for SIMIBI proteins head-to-head dimers (20–22, 30), where an extensive part of the protein homodimerization interface is stabilized by a network of hydrogen bonds and salt bridges among residues from both protein subunits, the sandwiched nucleotides and multiple coordinated water molecules. This complexation is coupled with conformational changes in the protein’s tertiary fold relative to apo-FleN, mostly by reorientation in helices α_2_ and α_5_ (30). As the same ATP-induced conformation is observed in the published FleQ^AAA+^-FleN complex, the authors proposed that ATP-binding and concomitant FleN dimerization is necessary for formation of the FleQ-compatible fold and FleQ recruitment (26). In support of this model, a FleN mutant carrying an alanine substitution in a key Walker A lysine responsible for nucleotide coordination in *trans* (FleN^K19A^) was found to no longer dimerize or hydrolyze ATP efficiently (at about 16% relative to wild-type FleN) and to no longer bind immobilized FleQ^AAA+^ in vitro (26, 30).

Surprisingly, the same mutant was found to inhibit significantly but not fully FleQ^FL^ ATPase activity in solution (at about 60% relative to wild-type when provided at saturating concentrations) (30), suggesting the preservation of both inhibitory FleQ-FleN interactions, as well as a catalytically functional FleQ-FleQ interface. We show here that upon coexpression with FleQ, FleN^K19A^ is still able to bind and copurify FleQ in the absence of ATP homologs and despite stringent washes prior to IMAC elution (*SI Appendix*, Fig. S2*C*). These data support a model where cellular ATP concentrations are sufficient for FleN remodeling and FleQ binding even in the absence of stable nucleotide complexation or FleN dimerization, and where FleN-dependent FleQ inhibition is mediated by a mechanism other than simple obstruction of the FleQ active site by its binding partner. Furthermore, as FleN is known to stabilize FleQ-promoter DNA binding (17, 27, 28), nucleoprotein complex formation might further stabilize interactions between FleQ and monomeric FleN in cellulo.

### Cryo-EM Structure of the c-di-GMP-Free FleN-FleQ^FL^ Assembly.

**Fig. 2.**
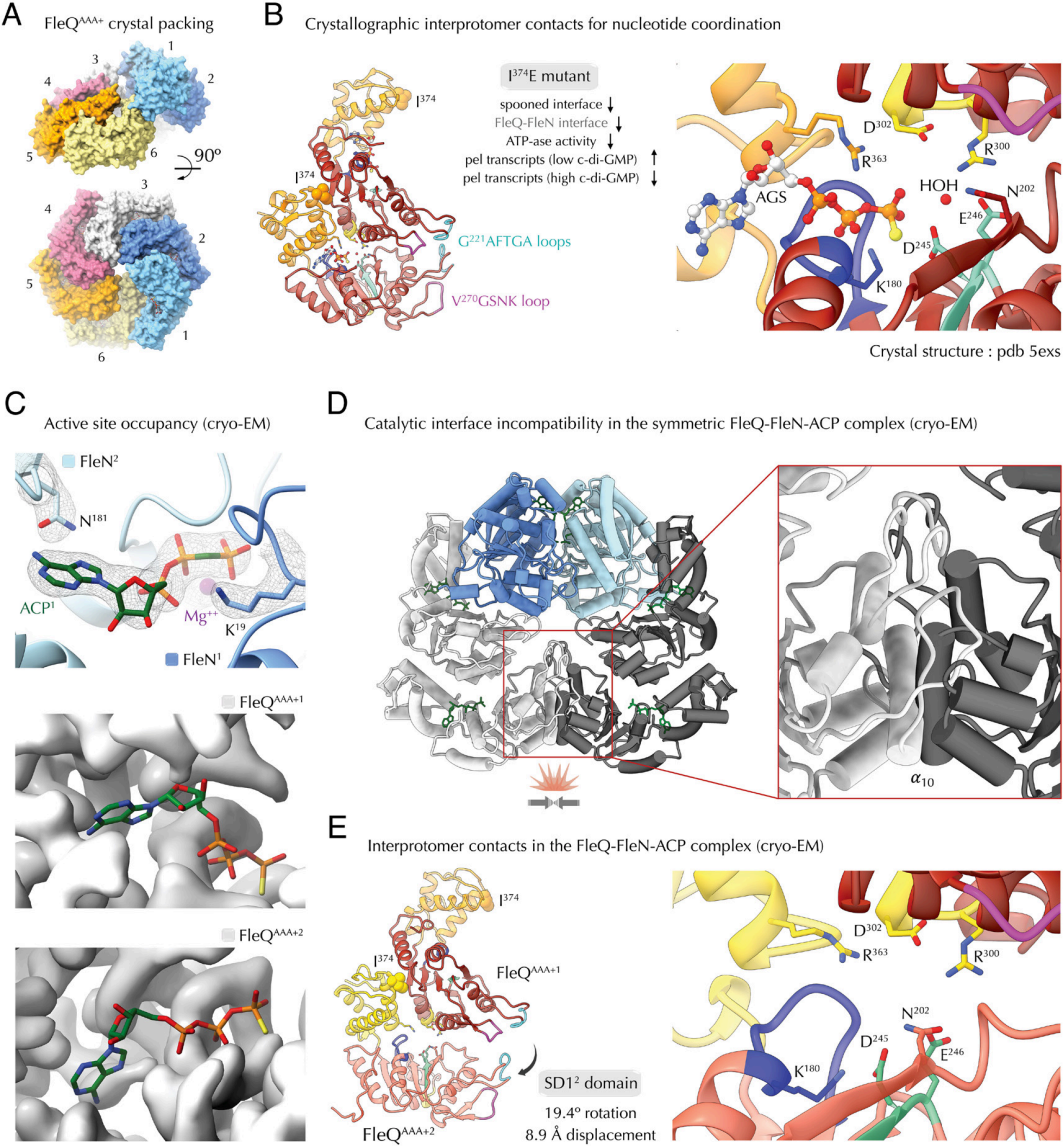
Determinants for FleQ ATPase activity and FleN-mediated FleQ inhibition. (*A*) Superhelical crystallographic packing of ATP-γ-S (AGS)–bound *P. aeruginosa* FleQ^AAA+^ compatible with the active hexameric assemblies of canonical bEBPs. (*B*) Crystallographic spooned FleQ contacts requisite for ATP hydrolysis. *Left*, a crystallographic dimer with coordinated ATP-γ-S (AGS) in the active site (11). The interface I^374^ residue is shown in spheres and reported physiological effects of the I^374^E mutation relative to wild-type FleQ are summarized (11). *Right*, a zoom-in of the nucleotide-binding pocket with key residues, the nonhydrolysable ATP-γ-S (AGS) and a catalytic water molecule shown. (*C*) Nucleotide occupancy in the FleQ-FleN-ACP complex. Top, AppCp (ACP) coordination by FleN showing the fitted nucleotide, a Mg^2+^ ion and some of the *cis* and *trans* coordinating residues in their corresponding electron densities. As the map is symmetric only one of the two ACP-bound pockets is shown. Below, lack of ACP coordination by the FleQ^AAA+^ domains, again shown for half of the symmetric assembly. The nucleotide-binding pocket was determined based on alignment of each FleQ^AAA+^ protomer with the crystal structure of ATP-γ-S (AGS)–bound FleQ (11), and the nucleotide is presented as sticks. The experimental cryo-EM density map is presented as surface in gray. (*D*) Incompatibility of the FleN-bridged cryo-EM structure with the catalytically competent FleQ-FleQ contacts. Alignment of a crystallographic FleQ^AAA+^-dimer onto each of the FleN-proximal FleQ^AAA+^ copies leads to significant steric clash between the SD1 modules of the peripheral FleQ^AAA+^ protomers. (*E*) A FleQ dimer as observed in the c-di-GMP-free FleQ-FleN structure solved by cryo-EM. SD1 displacement of the peripheral FleQ^AAA+^ copy relative to that of the crystallographic FleQ^AAA+^ dimer was calculated with PyMol (Schrödinger). A zoom-in of the empty nucleotide binding pocket and side chain orientations are shown on the *Right*.

To gain further insights into FleN-dependent FleQ regulation, we used single-particle cryo-EM and solved the solution structure of FleN-bound FleQ^FL^ in the absence of the second messenger c-di-GMP. We used the FleN^D48A^ mutant and included incubation with a nonhydrolysable ATP homolog in the purification procedure (AppCp, also referred to as ACP), to trap FleN in the sandwich dimer state due to binding but not hydrolysis of the nucleotide cofactor. Strikingly, the quaternary structure of the captured state features an overall C2 symmetry with unexpected 4:2 FleQ:FleN stoichiometry (Fig. 1 *C*–*E*). The core of the assembly is similar to the recently reported FleQ^AAA+^-FleN crystal structure, in which the nucleotide-bound FleN sandwich dimer is complexed on each side by a FleQ^AAA+^ domain (26) (*SI Appendix*, Fig. S1*B*). The latter features the same relative closure between its SD1 and SD2 modules, as well as partial obstruction of the active site by interaction with the C-terminal tail of FleN (26). Interestingly, each of these FleN-bound FleQ^AAA+^ copies are themselves complexed by a second FleQ^AAA+^ protomer in a conformation reminiscent of, but not identical to, the spooned intersubunit contacts observed in the crystal structures of c-di-GMP-free FleQ^AAA+^ and proposed to be essential for catalysis (11) (Figs. 1 *C*–*E* and 2). These peripheral FleQ^AAA+^ copies come into contact and likely stabilize the quaternary structure at the center of the assembly, underneath but without interfacing with the central FleN sandwich dimer. At each side at the periphery, the receiver domains of the two spooned FleQ protomers are found in the essential for FleQ function α_1_-mediated dimerized state (15), and the 4:2 stoichiometry is further stabilized by *trans* contacts between the SD2 of the peripheral FleQ^AAA+^ module and α_5_ from the receiver domain of the FleN-binding one (Fig. 1 *C*–*E*). Finally, despite the C-terminal HTH module being present in the copurified FleQ construct, it is not visible in the obtained electron density map. These results are similar to the previously reported crystal structure of c-di-GMP-bound FleQ^AAA+-HTH^ where only the ATPase modules are resolved in the density (11), suggesting high conformational variability. In the cryo-EM map a tubular density near the resolved C-terminal end of each peripheral FleQ^AAA+^ copy consistent with a predicted N-proximal AAA+-to-HTH linker helix suggests that FleQ^HTH^ dimerization likely occurs within pairs of spooned FleQ protomers on either side of the central symmetry axis rather than between FleQ protomers from opposite sides of the FleN sandwich dimer (Fig. 1*E*). Overall, both the FleQ^REC^ and FleQ^HTH^ domains are likely to assemble and function as dimers, which can explain the observed 4:2 stoichiometry where FleQ-FleN interactions disrupt higher-order FleQ oligomerization but not the dominant dimeric state (16).

### FleQ ATPase Regulation by Reversible FleN Dimerization and Spooned Interface Disruption.

As mentioned above, ATP hydrolysis by FleQ likely occurs in an oligomeric state similar to the one captured in the crystal structures of c-di-GMP-free FleQ^AAA+^ (Fig. 2 *A* and *B*). Importantly, it requires complexation between adjacent spooned protomers where conserved residues and motifs contribute to nucleotide and metal ion complexation, as well as catalytic water polarization for nucleophilic attack on the γ-phosphate (11) (Fig. 2*B* and *SI Appendix*, Fig. S3). Many of these residues act in *trans*, such as the conserved R^300^ from the classical arginine finger motif (R^300^xDL) (Fig. 2*B* and *SI Appendix*, Fig. S3). In the ATP homolog-bound FleQ^AAA+^ crystal structure R^300^ interacts with both the glutamate switch residue E^246^ from the water-activating Walker B motif in *trans* and with D^302^ from its own chain to keep the negative charge away from the triphosphate (11). This allows positioning of the *cis*-sensor II residue R^363^ in proximity to the γ-phosphate likely taking over the role of an arginine finger in bond polarization and hydrolysis (11) (Fig. 2*B*). In addition, the Walker A K^180^ and E^181^ involved in bond polarization and metal ion coordination, are stabilized by interactions with the nucleotide and two conserved arginines, post-Walker A R^185^ in *cis* and a conserved R^260^ in *trans* (11). Finally, ATP binding is stabilized by the REC-to-AAA+ domain linker by backbone interactions with the adenine base of the nucleotide, as well as by π-stacking interactions between linker R^144^ and a conserved R^334^ from SD2, stabilizing the latter to coordinate the adenine and ribose moieties in the active site pocket (11). Consistent with a model where hydrolysis requires activation between FleQ protomers in *trans*, an I^374^E mutant targeting the spooned interface without disrupting the ATP binding pocket per se was previously shown to inhibit ATPase activity in vitro and to derepress *pel* exopolysaccharide expression in cellulo in the absence of elevated c-di-GMP (Fig. 2*B*). Interestingly, as the mutation would target the observed FleQ-FleN interface preserved in the c-di-GMP-bound state as well (see below), the here-in presented structural data can also explain the failure of FleQ I^374^E to fully activate *pel* exopolysaccharide expression upon increase in cellular c-di-GMP (11).

In the c-di-GMP-free FleQ-FleN cryo-EM structure, the stabilizing nonhydrolysable ATP homolog AppCp can be found bound only to FleN but not to any of the complexed FleQ copies (Fig. 2*C*). Within the assembly, FleN-dependent dimerization renders preserving the catalytically competent spooned FleQ contacts described above impossible due to a steric crash between the SD1 modules of the peripheral FleQ protomers (Fig. 2*D*). This results in a 19.4° rotation and an approximately 9 Å displacement of the peripheral SD1 module as compared to the FleQ-FleQ interactions observed in the nucleotide-complexed crystal structures (11) (Fig. 2*E* vs. Fig. 2*B*). This leads to an open dimer interface where the σ^54^-interacting GAFTGA loops are split away, the REC-to-AAA+ domain linker curves into and R^144^ is flipped away from the ATP-binding pocket, the nucleotide-coordinating *trans*-R^363^ and Walker A K^180^ are no longer within proximity to coordinate the nucleotide’s γ-phosphate, and *trans*-R^300^ is displaced away from the Walker B motif (Fig. 2*E*).

**Fig. 3.**
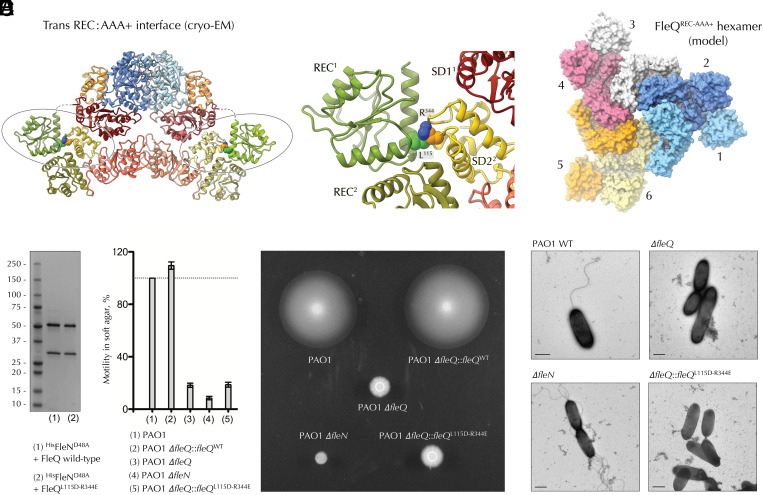
Intersubunit REC-AAA+ interactions necessary for FleQ activity. (*A*) The REC-AAA+ interface formed in *trans* between interacting FleQ protomers. *Left*, in the context of the FleQ-FleN complex; *Right*, zoom-in of the *trans* interface with the L^115^ and R^344^ residues shown as spheres. (*B*) Compatibility of the trans REC-AAA+ interface with higher-order FleQ oligomers based on the previously reported crystal contacts. (*C*) Copurification of wild-type FleQ and FleQ^L115D-R344E^ with ^His^FleN. (*D*) Motility in soft agar as compared among wild-type *P. aeruginosa* PAO1, a Δ*fleN* deletion strain, and a Δ*fleQ* strain complemented with wild-type or L^115^D-R^344^E mutant *fleQ* at its endogenous chromosomal locus. *Left*, results representative of 3 independent experiments with 2 biological replicates each. *Right*, a representative soft agar plate. (*E*) Typical flagellation of wild-type *P. aeruginosa* PAO1 as compared to the Δ*fleN*, Δ*fleQ*, and the *fleQ*^L115D-R344E^ mutant strains.

Finally, the presented c-di-GMP-free FleQ-FleN structure also provides insights into interactions between the essential for activity FleQ^REC^ and FleQ^AAA+^ domains. As mentioned above, the assembly reveals the requisite for FleQ activity REC domain dimerization (15), as well as a *trans* interface between the SD2 of the peripheral FleQ^AAA+^ module and α_5_ from the receiver domain of the FleN-bound FleQ protomer (Fig. 3*A*). We hypothesized that this interface—compatible with the formation of higher-order cooperative FleQ oligomers (Fig. 3*B*)—likely stabilizes the spooned FleQ oligomerization necessary for FleQ-mediated ATP hydrolysis and σ^54^-dependent gene expression activation. Consistent with this, a double FleQ^L115D-R344E^ mutant featured preserved expression, FleN binding, and copurification in recombinant context, but—when expressed from the original *fleQ* chromosomal locus in *P.* *aeruginosa*—showed severely disrupted motility in soft agar and no flagellar assembly, similarly to the Δ*fleQ* mutant (Fig. 3 *C*–*E*).

Together, the above observations support a model where FleN dimerization-dependent FleQ inhibition is not so much induced by obstructing nucleotide binding at the FleN-interacting FleQ protomer, but rather by breaking the catalytically relevant FleQ-FleQ contacts and preventing the formation of higher-order cooperative FleQ oligomers. Such a model is also compatible with the biochemical data discussed above of preserved FleQ interactions with monomeric FleN, the resulting dampened but not abolished FleQ hydrolytic activity upon such interactions, the actual stimulating effect of FleN on FleQ-DNA binding, and the FleQ ATPase activity switch-off upon FleN dimerization. Importantly, FleN-dependent ATP hydrolysis and associated dimer dissociation will simply enable spooned interface reconstitution and restore a basal FleQ hydrolytic activity without the need for FleQ-FleN or FleQ-DNA disassembly.

### FleQ^HTH^-DNA Consensus Interactions and Biofilm Repression in the Absence of c-di-GMP.

As mentioned before, the FleQ^HTH^ module has so-far evaded structural determination by both crystallographic and cryo-EM approaches. Previous work has demonstrated that the protein binds to ~22 base pair (bp)-long FleQ boxes featuring a central conserved region of 14 bp (17). The latter contains the inverted repeats GTCAAT and ATTGAC, which are separated by two nucleotides, mostly A or T (17) (*SI Appendix*, Fig. S4). The pseudopalindromic architecture of this binding consensus suggests that FleQ^HTH^ binds as a symmetrical dimer. To gain insights into promoter recognition, we used the deposited AlphaFold tertiary structure model for full-length FleQ (31) and used the extracted HTH fragment as a search model for structurally similar proteins across the Protein Data Bank using the Dali server (32). The top hits in the results corresponded to structures of the DNA-binding HTH domain of the Factor for Inversion Stimulation, or FIS protein, which binds to weakly related DNA sequences to regulate DNA replication from the OriC, DNA inversion events, and transcription of a diverse set of rRNA and virulence genes in *Escherichia coli* and/or *Salmonella enterica* serovar Typhimurium (33). In particular, FIS binds as a homodimeric molecule to a loose 15-nucleotide consensus sequence introducing a bend in the DNA backbone (34, 35) (*SI Appendix*, Fig. S4*A*). Interestingly, the top DALI hit was for a DNA-bound FIS^HTH^ dimer with rmsd of 0.57 Å over the aligned atoms. We thus modeled a DNA-bound FleQ^HTH^ dimer using the FIS^HTH^ assembly as a starting point and the 14-nucleotide double-stranded FleQ-binding consensus (*SI Appendix*, Fig. S4 *B–D*). The resulting model confirms that a single pseudopalindromic FleQ-box is indeed likely to accommodate a symmetric FleQ^HTH^ dimer. In the modeled assembly, a conserved tandem of arginines (R^467^R) could potentially contribute to sequence specificity by coordinating the 5’-GxC ends of the consensus, whereas the central AT-rich tract is likely to adopt a narrow minor groove and lead to an overall bent architecture (36) (*SI Appendix*, Fig. S4 *B–D*).

It remains uncertain why FleQ is a weak DNA binder on its own and instead requires FleN for efficient nucleoprotein complex formation, but a likely explanation is that FleN releases FleQ^HTH^ from an autoinhibited conformation. The observation that each FleQ^HTH^ dimer would bind to a FleQ box in promoter DNA can also explain the repressive role of FleQ-FleN complexes on adherence factor expression in the absence of c-di-GMP (17, 29). As mentioned above, unlike flagellar operons that typically feature a single, upstream FleQ box in their promoters, most biofilm-related genes have a tandem of FleQ-binding sequences often close to or overlapping with additional regulatory regions (Fig. 1*B*) (17). The above-presented FleQ-FleN structure is compatible with a model where each of the FleN-bridged FleQ dimers would bind a FleQ box within the promoter and could thus lead to large-scale DNA bending stabilized by the central contacts between the SD1 modules of the peripheral FleQ copies and by the intrinsic DNA-bending propensity of the FleQ^HTH^ tandems themselves. Such stabilization could further decrease FleN’s weak ATPase activity, dimer disassembly, and/or nucleotide recycling, thus repressing transcription initiation via promoter occlusion (see also summary figure below).

### Cryo-EM Structure of the c-di-GMP-Bound FleN^D48A^-FleQ^REC-AAA+^ Assembly and Insights into Promoter Control.

**Fig. 4.**
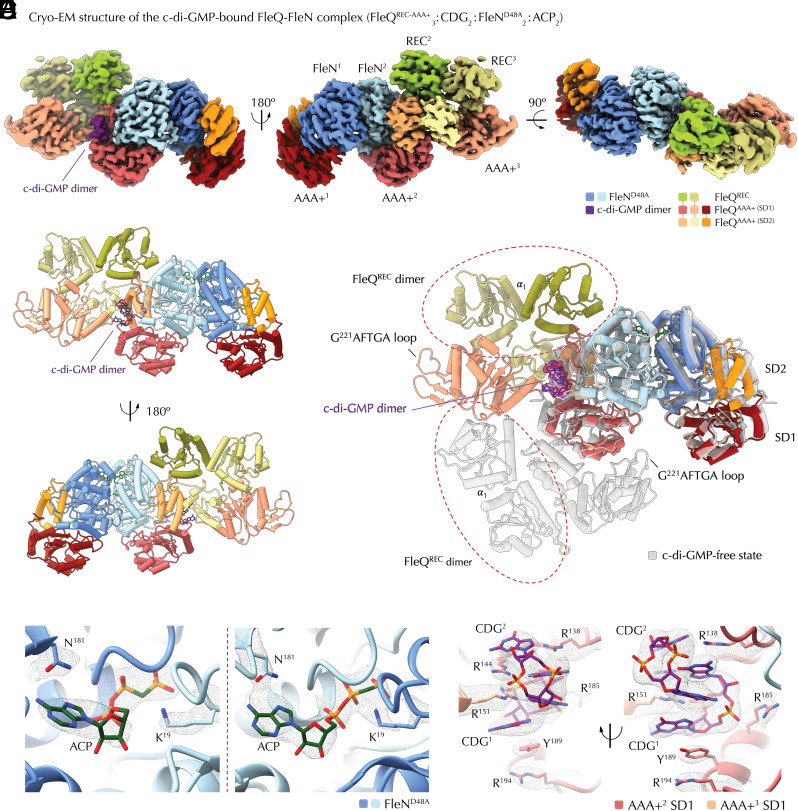
Cryo-EM structure of the c-di-GMP-free FleQ-FleN complex. (*A*) Segmented, color-coded electron density map of the purified FleQ^REC-AAA+^-FleN^D48A^ complex bound to the nonhydrolysable ATP homolog AppCp (ACP) and c-di-GMP (CDG) as resolved by cryo-EM. (*B*) Cartoon representation of the solved FleQ^REC-AAA+^_3_:CDG_2_:FleN^D48A^_2_:ACP_2_ cryo-EM structure, color-coded as in *A*. Intercalated dimeric c-di-GMP is shown as sticks in purple. (*C*) Overlay of the corresponding protomers between the c-di-GMP-free (in gray) and c-di-GMP-bound structures. C-di-GMP is represented in its corresponding electron density at the FleQ-FleQ interface. (*D*) Coordination of ACP by the FleN protomers. (*E*) Two views of the intercalated c-di-GMP dimer and coordinating residues as sticks in the corresponding experimental electron densities. Additional residues from the dinucleotide binding pocket and the related FleQ-FleQ interface are shown in Fig. 5.

**Fig. 5.**
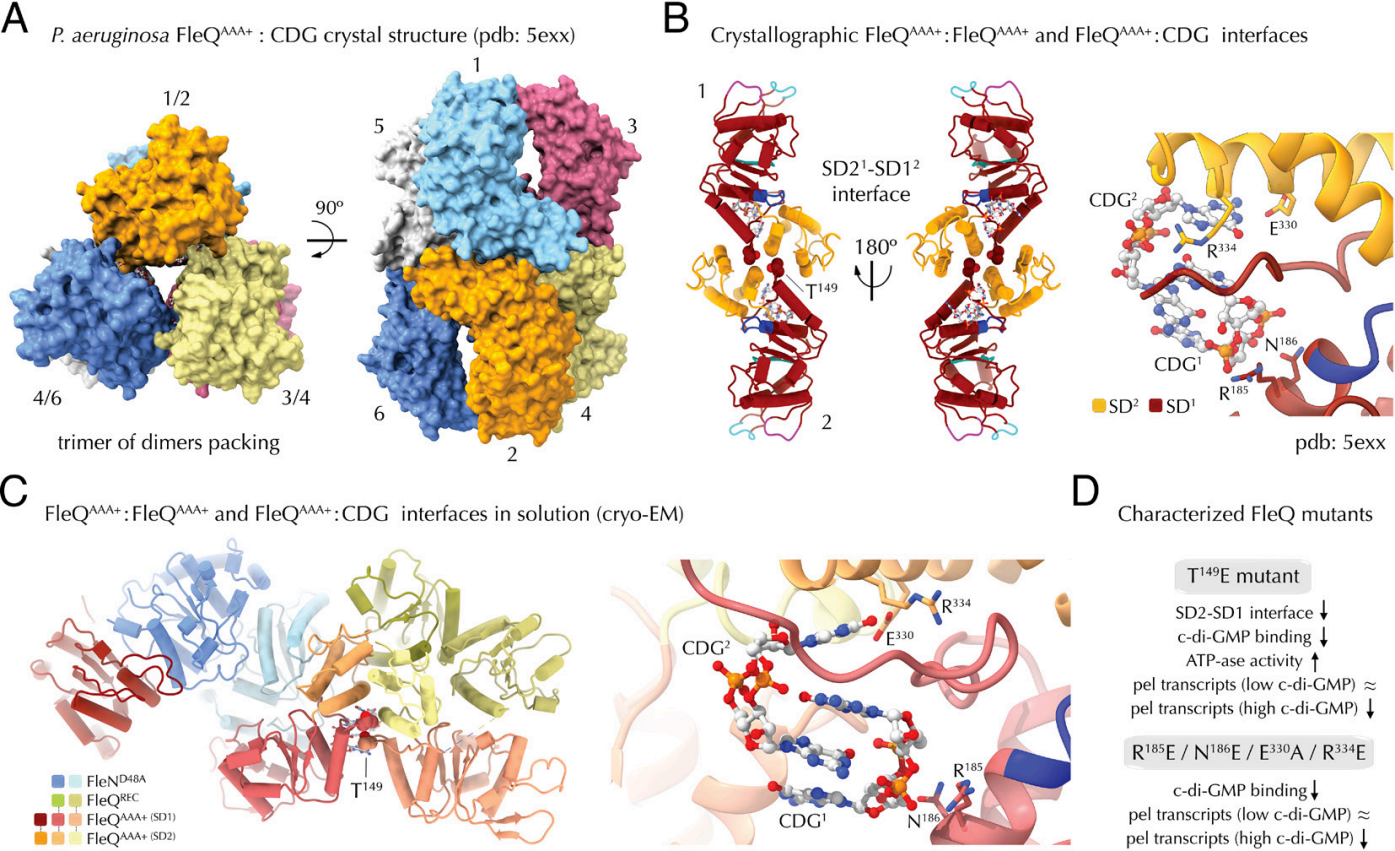
C-di-GMP-dependent interfaces. (*A*) Trimer-of-dimers crystallographic packing of c-di-GMP-bound FleQ^AAA+^. (*B*) Crystallographic interfaces. *Left*, a crystallographic c-di-GMP-bound FleQ^AAA+^ elongated dimer. The interface T^149^ residue is shown as spheres. *Right*, zoom-in of the intercalated c-di-GMP dimer with functionally characterized pocket residues shown as sticks. (*C*) Corresponding residues in the cryo-EM structure. *Left*, cartoon representation of the c-di-GMP-bound cryo-EM structure with the interface T^149^ residue shown in spheres. *Right*, positioning of the functionally characterized residues around the c-di-GMP binding pocket. (*D*) Summary of the previously reported physiological effects of interface and c-di-GMP-binding FleQ mutants relative to wild-type.

Given the central role of c-di-GMP in motile-to-sessile lifestyle transition, we next pursued to decipher the effects of dinucleotide binding on FleQ-FleN complex formation (Figs. 4 and 5). Previous research has shown that c-di-GMP binding to FleQ inhibits its ATPase activity and flagellar gene expression and converts the protein from repressor into activator of biofilm-related gene clusters (16, 29). The crystal structure of c-di-GMP-bound FleQ^AAA+-HTH^ revealed drastic conformational changes, where the resolved AAA+ domains arrange into an atypical hexamer (11) (Fig. 5*A*). The assembly can be viewed as a symmetric trimer of elongated tail-to-tail dimers where the main intersubunit contacts are established by residues from the centrally positioned SD2 domains and the loops preceding SD1 helices α_6_, whereas c-di-GMP is complexed as an intercalated dimer per FleQ protomer resulting in a 1:2 protein-to-ligand stoichiometry (11) (Fig. 5 *A* and *B*). The REC-to-AAA+ domain linker is partially inserted into the ATP-bound pocket and the c-di-GMP dimer is stabilized by a number of arginine residues, including R^144^, R^185^, and R^334^ that also participate in ATP coordination and hydrolysis as discussed above. Consistent with the crystal structure contacts, mutations in either R^185^ or R^334^ were shown to disrupt both c-di-GMP complexation and FleQ ATPase activity, whereas multiple additional mutations validated the functional roles of c-di-GMP binding and intersubunit FleQ contacts (11).

Nevertheless, the observed quaternary structure of the crystallized FleQ^AAA+-HTH^ construct (11) is not compatible with the FleQ-FleN contacts observed in the crystal structure of the FleN-FleQ^AAA+^ complex (26) or the cryo-EM structure of the c-di-GMP-free FleN-FleQ state presented above. We therefore resorted to single-particle cryo-EM to again gain insights into the actual c-di-GMP-bound assembly in solution (Fig. 4 *A*–*C*). Due to higher protein yields and increased complex stability determined empirically, we chose again the FleN^D48A^ variant for FleN and an HTH-truncated construct for otherwise wild-type FleQ (FleQ^REC-AAA+^).

Importantly, the resulting 3D reconstruction features the same FleQ^AAA+^-hugged FleN dimer as observed crystallographically (26, 30) (*SI Appendix*, Fig. S1*B*) and by cryo-EM in the absence of c-di-GMP (Fig. 3), indicating that dinucleotide-sensing effects are mostly determined by the conformational and functional plasticity of FleQ itself. However, the similarities between the two presented cryo-EM structures stop here. First, the global architecture of the complex is asymmetric with a 2:3 FleN:FleQ stoichiometry and overall increased flexibility (Fig. 4 *A*–*C*). One of the FleN protomers is bound to a single FleQ subunit with reduced occupancy, resulting in slight distortion of the electron density map and loss of signal for the N-terminal receiver domain (Fig. 4*A*). The other FleN protomer is bound to a dimer of c-di-GMP-complexed FleQ subunits, in a conformation drastically different from the previously captured crystalline state (11) (Fig. 4 *A*–*C*).

Whereas FleN remains bound to the nonhydrolysable AppCp, c-di-GMP is bound as a single intercalated dimer at the interface between the dimerizing FleQ protomers (Figs. 4 and 5*B*), i.e., in a 1:1 c-di-GMP:AAA+ stoichiometry as opposed to the 2:1 stoichiometry observed in the crystals (11). The FleN-bound AAA+ module mediates the majority of the contacts with the dinucleotide dimer, in a conformation very similar to the individual FleQ^AAA+^-c-di-GMP_2_ assembly from the crystallized state (11) (rmsd of 1.2 Å over all atoms, with slight closure between the SD1 and SD2 domains). C-di-GMP occupies an almost identical pocket and is coordinated primarily by several arginine (backbone and side-chain interactions with R^138^, R^144^, R^185^, and R^334^ in *cis* and R^151^ in *trans*) and glutamate (Glu^330^ in *cis* and Glu^332^ in *trans*) residues, whereas the REC-to-AAA+ domain linker inserts deep into the ATP-binding pocket to then thread onto the surface of the interacting FleN protomer (*SI Appendix*, Fig. S5*A*).

Perhaps most surprisingly, rather than engage in an elongated symmetrical dimer as in the crystal structure (11) (Fig. 5 *A* and *B*), the second FleQ^AAA+^ module establishes asymmetric tail-to-tail contacts again mediated by SD2 and the loop preceding SD1 α_6_. It is important to note that previously performed in vitro and in vivo functional analyses of FleQ mutants targeting the observed FleQ-FleQ and FleQ-c-di-GMP contacts in crystallo (11) actually validate the cryo-EM structure presented here due to the overall preserved dinucleotide binding site and buried FleQ surface areas between the crystallized and cryo-EM-derived c-di-GMP-bound states (Fig. 5). For example, T^149^ occupying a central position at the SD1-SD2 interface in the crystallized FleQ^AAA+^-c-di-GMP complex is again nestled at the interface between the dimerized FleQ^AAA+^ protomers and the intercalated dinucleotide within the larger c-di-GMP-bound FleQ-FleN complex (Fig. 5 *B*–*D*).

**Fig. 6.**
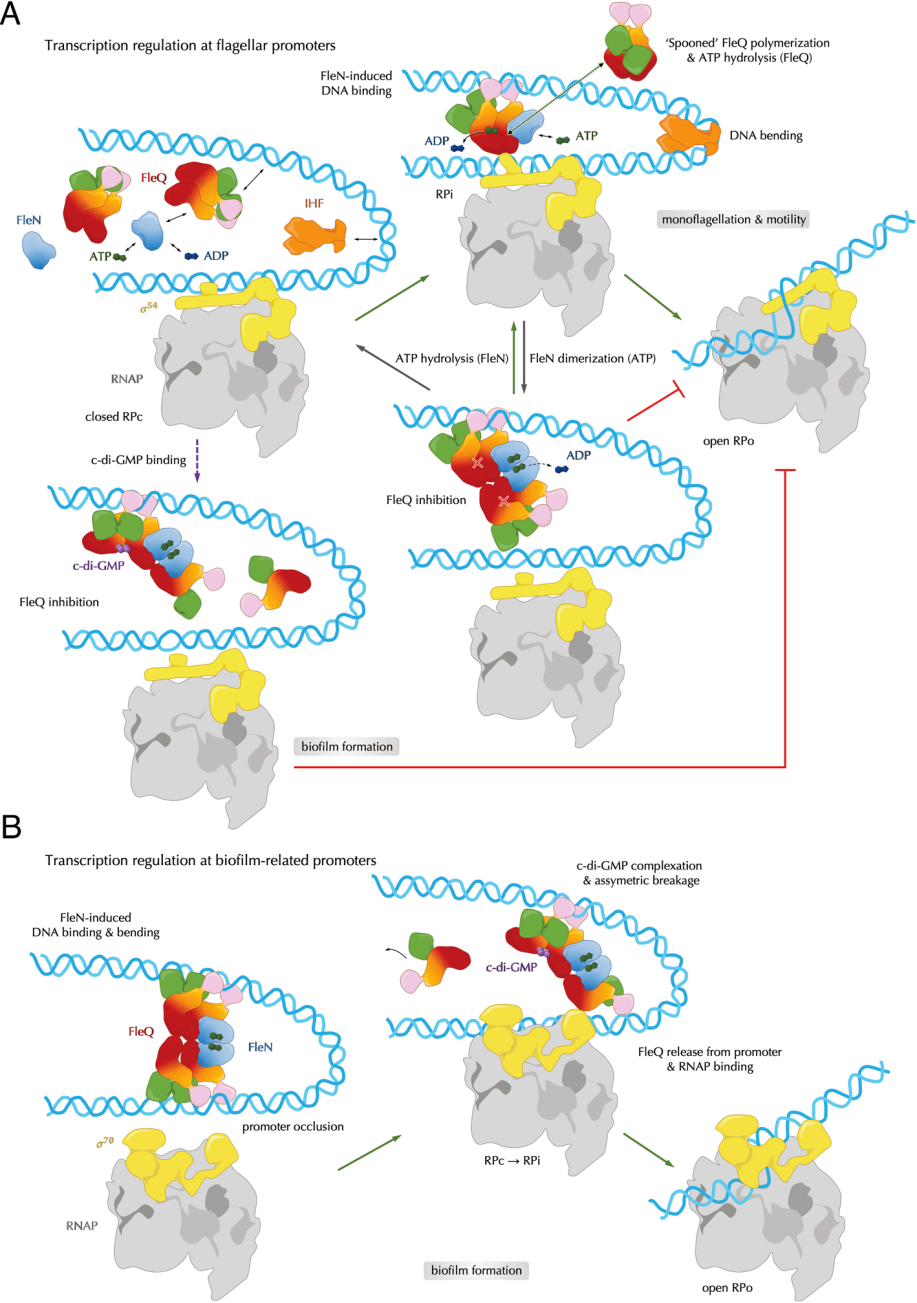
Functional model of FleQ-FleN roles at flagellar and biofilm-related promoters. (*A*) FleQ-FleN control of flagellar genes. RP: RNA polymerase (RNAP)-promoter DNA complexes (c: closed, i: intermediate, and o: open); IHF: Integration Host Factor. Monoflagellation is achieved between fine-tuning ATP-dependent FleQ hydrolysis and σ^54^-dependent transcription activation vs. ATP-dependent FleN dimerization and FleQ inhibition. FleN stimulates DNA binding and FleQ spooned oligomerization-dependent RPc-RPi-RPo transition (*SI Appendix*, Fig. S5*C*), whereas FleN dimerization inhibits RPi-RPo transition and locks FleQ dimers into a catalytically incompetent state. C-di-GMP binding prevents both RPi and RPo formation. (*B*) FleQ-FleN control of biofilm-related genes. Duplicate promoter boxes cause FleN-dependent DNA binding and bending, thus preventing RPi and RPo formation via promoter occlusion. C-di-GMP binding causes asymmetric complex disassembly, promoter release, and σ^70^-dependent transcription activation (RPi and RPo formation).

In the cryo-EM structure, the REC domain of the peripheral FleQ protomer is now shifted to nestle between the SD1 and SD2 modules, with its linker to the AAA+ domain not fully resolved but likely threading through the ATP binding pocket (*SI Appendix*, Fig. S5*B*). It further engages in the atypical, α_1_-mediated dimerization contacts with the REC domain of the neighboring subunit (15), which in turn is found atop the latter’s SD2 module (Fig. 4*C*). Again, the two cryo-EM structures presented here provide direct blueprints for the involvement of the noncanonical receiver domain interface in both biofilm formation and flagellar motility, as deduced previously from the crystal structure of the isolated FleQ^REC^ module and corroborated by phenotypic analyses (15). The observed c-di-GMP-bound assembly also provides further insights into dinucleotide-dependent inhibition of FleQ’s ATPase activity: not only are the ATP-binding pockets occupied by FleN’s C-terminal tail and FleQ’s REC-to-AAA+ domain linkers, but the tail-to-tail contacts and REC domain reorientation preclude any possibility for hydrolysis-competent spooned contacts or for further oligomerization in both the central and peripheral FleQ protomers. It is also important to note that prior to cryogrid preparation the complex was incubated with both c-di-GMP and the nonhydrolysable ATP homolog AppCp, where the latter was provided in a 50-fold excess (200 μM AppCp vs. 4 μM c-di-GMP). Together, these data demonstrate that physiological concentrations of c-di-GMP (37), which are ordinarily orders of magnitude lower than those of intracellular ATP, are sufficient to break the spooned FleQ contacts, inhibit ATPase activity, and consequently repress flagellar motility. Furthermore, the preserved FleN-FleN and FleN-FleQ contacts suggest that in the c-di-GMP-bound state both interfaces can form with higher affinity than the spooned FleQ-FleQ one, thus leading to asymmetric complex disassembly and leaving a single FleN-bound FleQ dimer bound to the promoter DNA. In the context of the *pel* operon and other biofilm-related clusters where a symmetric c-di-GMP-free FleQ-FleN complex is proposed to simultaneously bind two FleQ boxes and inhibit transcription via DNA looping, such disassembly can lead to promoter relaxation and ATP- and σ^70^-dependent transcription initiation the details of which are yet to be uncovered (Fig. 6).

### An Integrated Model of FleQ-FleN-Dependent Transcription Regulation.

FleQ is the first transcription factor identified to directly regulate motility and extracellular matrix production in response to the second messenger c-di-GMP (28). This makes the protein and its regulators an interesting target in the quest to disrupt both acute and chronic infection strategies employed by the opportunistic pathogen *P. aeruginosa*, as well as by additional species featuring the conserved FleQ-FleN regulatory tandem (*SI Appendix*, Fig. S3).

To better understand how FleQ-FleN interactions happen in a physiological context, we carried out in cellulo complex reconstitution using different truncated and mutant variants and determined solution cryo-EM structures of FleQ-FleN complexes in the presence of a nonhydrolysable ATP homolog (AppCp), with or without c-di-GMP. We show that stable complex formation requires the N-terminal FleQ receiver domain and not necessarily FleN dimerization. In light of the results presented here and the wealth of published biochemical data discussed above, we propose that at flagellar promoters transient interactions between monomeric FleN and spooned FleQ dimers stimulate promoter DNA recognition and complex stabilization, likely followed by further catalytically competent FleQ oligomerization, σ^54^-RNAP binding and FleQ ATPase-dependent RPi-to-RPo conversion. It is important to note that while FleN monomer binding is compatible with FleQ superhelical hexamerization (*SI Appendix*, Fig. S5*C*), further studies are necessary to visualize the specific stoichiometry and interactions in the context of the nucleoprotein transcription initiation complex. The c-di-GMP-free FleQ-FleN structure presented here integrates both FleQ^AAA+^-FleN and FleQ^REC^-FleQ^REC^ contacts reported previously (15, 26) but reveals a catalytically incompetent 4:2 FleQ:FleN assembly where FleQ hydrolytic activity is inhibited by both disruption of the spooned dimer interface and prevention of higher-order FleQ oligomerization by dimerized FleN. Subsequent ATP-hydrolysis cycles by FleN would allow reconstitution of cooperative FleQ contacts necessary for basal transcription activation, without necessarily disrupting protein–protein and actually stimulating protein–DNA interactions (Fig. 6*A*).

Conversely, at biofilm-related operons, the symmetric quaternary complex can simultaneously bind to the duplicate FleQ consensus boxes (17) and repress transcription through DNA bending and promoter occlusion (Fig. 6*B*). The c-di-GMP-bound state reveals both a dominant role for the dinucleotide in inhibiting FleQ ATPase activity and asymmetric breakage and reconfiguration of the quaternary complex (Fig. 6). As a result, one part of the assembly will remain devoid of FleN and likely release from the promoter DNA, whereas the other remains FleN- and consequently DNA-bound and in the case of biofilm-promoting genes can likely interface with the σ^70^-RNAP holoenzyme, as supported by earlier functional studies (17, 29). Future work is needed to determine how FleQ’s conformational plasticity and drastically different c-di-GMP-free and c-di-GMP-bound assemblies allow it to induce transcription via direct interactions with either σ^54^ or σ^70^ at the respective flagellar or biofilm-related promoters.

## Materials and Methods

The experiments were not randomized, and the investigators were not blinded during experimental design, execution, or outcome assessment.

### Bacterial Strains.

Plasmids for heterologous protein expression (see below) were propagated in and isolated from *E. coli* DH5α cells using Lennox LB and LB-agar media supplemented, as applicable, with the following concentrations of antibiotics: 100 μg/mL ampicillin for pProEx-Htb variants and 40 μg/mL kanamycin for pRSFDuet1*. All recombinant protein expression for structural and in vitro biochemical studies was carried out in *E. coli* BL21 Star^TM^ (DE3) cells using liquid TB (Terrific Broth) media, supplemented with the appropriate antibiotics (see below). Engineering of *P. aeruginosa* PAO1 mutant strains is described in detail below.

### Recombinant DNA Techniques.

Recombinant DNA manipulations were carried out using standard protocols for PCR, molecular cloning, transformation, and DNA analysis. Coding regions for FleQ and FleN variants were amplified using high-fidelity Phusion DNA polymerase (New England Biolabs) and *P. aeruginosa* PAO1 genomic DNA as a template and inserted via digestion/ligation cloning into isopropyl β-D-1-thiogalactopyranoside (IPTG)-inducible expression vectors with custom-modified multiple cloning sites (MCS) (*SI Appendix*, Table S1). Point mutations and MCS modifications were performed using inverse PCR-based protocols and mutation-specific oligonucleotides as primers, and PCR products were treated with DpnI restrictase for template DNA digestion prior to ligation and transformation. All restriction enzymes, T4 polynucleotide kinase, T4 DNA ligase, and calf intestinal phosphatase (Quick CIP) used in cloning were purchased from New England Biolabs. Propagated and purified recombinant vectors and introduced mutations were verified by DNA-sequencing and IPTG-inducible protein expression, where applicable.

### Protein Expression and Purification.

The coding regions corresponding to *P. aeruginosa* FleQ^FL^ and FleN^FL^ were PCR-amplified and introduced into a modified pProEx-Htb expression vector, in order to yield IPTG-inducible variants carrying N-terminal hexahistidine (His_6_) tags cleavable by the human rhinovirus 3c (HRV3c) protease (Fle^His^Q^FL^ and Fle^His^N^FL^). In parallel, a standard pRSF-Duet1 vector was PCR-amplified with primers CAT ATG GGA TCC CAT GGT ATA TCT CCT TAT TAA AG and CTC GAG GCG GCC GC A TAA TGC TTA AGT CGA ACA GA to remove the hexahistidine tag-coding region and yield custom-modified pRSFDuet1* (24). The latter was used in BamHI/NotI restrictase-mediated cloning to insert the DNA sequence corresponding to full-length untagged FleQ and FleN for coexpression with pProEx-Htb-encoded FleN and FleQ variants, respectively. Whereas we worked almost exclusively with the full-length FleN sequence, we studied several truncated FleQ constructs, namely FleQ^1-139^ covering the FleQ receiver domain (FleQ^REC^), FleQ^1-394^ covering the receiver and ATPase domain tandem (FleQ^REC-AAA+^), FleQ^138-394^ covering the isolated ATPase domain (FleQ^AAA+^), FleQ^1-490^ covering the full-length protein (FleQ^FL^), and finally a variant missing the last 13 amino acids based on previously published stable constructs (FleQ^FL*^) (11).

For recombinant protein expression, all expression vectors were freshly transformed into chemically competent *E. coli* BL21 Star^TM^ (DE3) cells and plated onto antibiotics-supplemented LB-agar plates (100 μg/mL ampicillin, 40 μg/mL kanamycin or a combination of 70 μg/mL ampicillin + 30 μg/mL kanamycin for coexpressed vectors). Resultant colonies were then inoculated and grown aerobically at 37 °C in TB medium supplemented with the appropriate antibiotics as above. At a cell optical density corresponding to light absorbance of 0.8 to 1.0 at 600 nm wavelength (OD_600_), the cells were moved to 17 °C and overnight protein expression was induced by the addition of IPTG at a final concentration of 0.7 mM. After 16 h, the cells were harvested by centrifugation (20 min at 4,000 g and 4 °C) and resuspended in the appropriate lysis buffer.

For initial detection of FleN-interacting FleQ fragments, pProEx-Htb-cloned wild-type and mutant FleN or FleQ constructs were cotransformed with pRSFDuet1*-cloned FleQ and FleN ones in various combinations. Following expression culture growth and overnight IPTG induction, the collected cells were resuspended in ice-cold lysis buffer containing 20 mM 4-(2-hydroxyethyl)-1-piperazineethanesulfonic acid (HEPES) pH 8.0, 120 mM NaCl, 19 mM Imidazole pH 8.0 and 1 tablet/50 mL c*O*mplete protease inhibitors (Roche). Cells were then lysed by sonication, and the debris was removed by centrifugation at 40,000 g for 1 h at 4 °C. The cleared lysates were loaded onto buffer-washed Talon Superflow^™^ resin (GE Healthcare) at approximately 0.5 to 1 mL of resin per liter of culture, and following a 15-min incubation and gravity flow, the resin was washed with over 40 column volumes of IMAC wash buffer A (protease inhibitor-free lysis buffer as above). Retained proteins were then eluted in a single step with IMAC elution buffer B (IMAC buffer A supplemented with 200 mM Imidazole at pH 8.0) and visualized by denaturing sodium dodecyl sulfate–polyacrylamide gel electrophoresis (SDS-PAGE).

For purification of the c-di-GMP-free FleQ-FleN complex, pProEx-Htb-cloned full-length FleN^D48A^ was cotransformed with pRSFDuet1*-cloned wild-type FleQ^FL^. After overnight recombinant expression as above, the cells were lysed in empirically optimized lysis buffer containing 20 mM HEPES pH 8.0, 250 mM NaCl, 19 mM Imidazole pH 8.0, 2 mM MgCl_2_, 5% glycerol, 1 tablet/50 mL c*O*mplete protease inhibitors (Roche) and 1 mg/mL chicken egg lysozyme (Sigma-Aldrich). Cells were lysed by sonication and the lysate was cleared by centrifugation as above and loaded onto the Talon Superflow™ resin (GE Healthcare). The IMAC wash buffer A contained 20 mM HEPES pH 8.0, 250 mM NaCl, 19 mM Imidazole pH 8.0, 2 mM MgCl_2_, and 2% glycerol, and the IMAC elution buffer B was similarly supplemented with 200 mM Imidazole. Following elution, the collected sample was supplemented with AppCp [Adenosine-5′-[(β,γ)-methyleno]-triphosphate, also referred to as AMPPCP or ACP: a nonhydrolysable ATP homolog (Jena Bioscience)] at a final concentration of 100 μM. Without tag cleavage, the eluted sample was concentrated to 2.5 mL volume and desalted using a disposable PD-10 column with buffer containing 20 mM HEPES pH 8.0, 250 mM NaCl, 12 mM Imidazole pH 8.0, 2 mM MgCl_2_, and 5% glycerol. Following elution, the sample was reconcentrated and injected onto a Superdex 200 Increase 10/300 GL size exclusion column equilibrated with gel filtration buffer (20 mM HEPES pH 8.0, 250 mM NaCl, 2 mM MgCl_2_, and 2% glycerol). Following elution, the protein distributions were analyzed for purity and complex formation by SDS-PAGE, and the protein fractions of interest were then concentrated onto an Amicon Ultra centrifugal filter (50 kDa cutoff; Millipore) and used for cryo-EM grid preparation. For the latter, the samples were flash-frozen in liquid ethane using glow-discharged UltrAufoil R1.2/1.3 grids and a Vitrobot Mark IV device (ThermoFisher Scientific) prechilled to 4 °C at 100% chamber humidity. Excess proteins were aliquoted and flash frozen for storage at −80 °C.

For purification of the c-di-GMP-bound FleQ-FleN complex, pProEx-Htb-cloned full-length FleN^D48A^ was cotransformed with pRSFDuet1*-cloned wild-type FleQ^REC-AAA+^. Protein expression and IMAC purification were carried out as for the c-di-GMP-free complex above. Following IMAC elution, the collected sample was mixed with AppCp and c-di-GMP (Jena Bioscience) at final concentrations of 100 μM and 5 μM, respectively, and the hexahistidine tag on FleN^D48A^ was cleaved by overnight incubation at 4 °C with purified His-tagged HRV3c protease. On the next day, the sample was concentrated and subjected to gel filtration in the presence of c-di-GMP (gel filtration buffer: 20 mM HEPES pH 8.0, 250 mM NaCl, 2 mM MgCl_2_, 2% glycerol, 2 mM DTT, and 2 μM c-di-GMP). Elution fractions were analyzed for purity and complex formation, and AppCp and c-di-GMP were added to final concentrations of 200 μM and 4 μM, respectively, before cryo-EM grid preparation as above.

### SDS-PAGE.

Protein fractions were analyzed by standard denaturing SDS-PAGE electrophoresis using 4 to 20 % gradient mini-gels (Bio-Rad), Expedeon InstantBlue™ Coomassie stain, and a Gel Doc™ EZ system (Bio-Rad) for Coomassie stain visualization.

### Cryo-EM and Single-Particle Analysis.

Preliminary datasets on both the c-di-GMP-free and c-di-GMP-bound FleQ-FleN complexes were collected on the Elsa Talos Arctica transmission electron microscope (Thermo Fisher Scientific) at the European Institute of Chemistry and Biology (IECB Bordeaux, France) operated at 200 kV and equipped with a Gatan K2 Summit direct electron detector (DED) operated with SerialEM in counting mode. For each sample, about two thousand movies were collected with a total electron dose of ~50 e^−^/Å^2^ and a corrected pixel size of 0.93 Å^2^. The movies were motion- and CTF-corrected using MotionCor2 (38) and Gctf (39), respectively, after which all micrograph processing was continued in cryoSPARC v3 and v4 (40). Particles were autopicked using the software’s “Blob picker” function, and after 2D classification, the best-resolved representative views (2D class averages) were used as templates for new rounds of template-based autopicking, 2D classifications, and data clean-up. Particles belonging to 2D classes with well-resolved secondary structure features were selected and used for ab-initio model generation with three 3D classes and after heterogeneous refinement, the best class was subjected to non-uniform refinement yielding 3D models with resolution of approximately 4.5 Å for both states. A total of ~117,000 and ~140,000 particles were used for the final models in the c-di-GMP-bound and -free states, respectively. Importantly, due to the peculiar elongated and flat shape of both structures, as well as conformational flexibility of the assembled complexes, the datasets featured a significant bias in the orientation of the aligned particles yielding to density deformation more evident for the c-di-GMP bound complex.

To improve the resolutions and reliably interpret the 3D electron density maps, we resorted to data collection on optimized samples using 300 kV Titan Krios microscopes equipped with K3 DEDs and Gatan GIF Quantum LS energy filters. For structure resolution of the c-di-GMP-free FleQ^FL^-FleN^D48A^ complex, a dataset of 21,223 movies with total electron dose of 51.5 e^−^/Å^2^ and pixel size of 0.66 Å^2^ was collected at the CM01 Titan Krios at the ESRF Grenoble (41). Movies were motion- and CTF-corrected as above and a total of 17,099 exposures with resolutions better that 4.4 Å and a defocus range of 0.3 to 2.5 μm were retained for further processing. Template-based autopicking was performed on a subset of ~5,300 micrographs using the best 2D class averages from the preliminary dataset collected on the IECB Talos Arctica and, following parameter optimization and 2D classification, well-resolved 2D classes were used for template-based autopicking on the full dataset. A total of 4,124,200 particles were initially extracted and, following 2D classification, well-resolved class averages containing a total of 562,273 particles were retained and input for ab-initio model generation with three 3D classes, followed by heterogeneous refinement. 404,228 particles belonging to the best-resolved class were re-extracted and input for nonuniform refinement with C2 symmetry. The resultant map was sharpened using the integrated Deep EMhancer tool (42) and used for rigid body fitting of structures of individual FleQ and FleN domains in Chimera, followed by model building and regularization in Coot (43) and refinements in Phenix (44) and Namdinator (45).

For structure resolution of the c-di-GMP-bound FleQ^REC-AAA+^-FleN^D48A^ complex, 14,640 movies with a total electron dose of 50.3 e^−^/Å^2^ and pixel size of 0.645 Å^2^ were collected at the EMBL Imaging Center in Heidelberg, Germany and, following motion- and ctf-correction as above, 14,268 exposures with defocus range between 0.8 and 2 μm and resolutions better than 4 Å were retained for further analysis. Particle picking was performed using 2D class averages from the preprocessed Talos Arctica dataset, and following multiple rounds of 2D classification, ab-initio model generation, and heterogeneous refinement, the best resolved class corresponding to 1,283,731 particles was input for non-uniform refinement. As the resultant map presented obvious overfitting artifacts due to overrepresented orientations and intrinsic flexibility of the complex, the particles were downsampled to pixel size 1.419 Å^2^ and subjected again to nonuniform refinement. The resultant map was sharpened with Deep EMhancer (42) through the cryoSPARC interface and used for rigid body fitting of structures of individual FleQ and FleN domains in Chimera, followed again by model building and regularization in Coot (43) and refinements in Phenix (44) and Namdinator (45).

Coordinate refinement statistics are summarized in *SI Appendix*, Table S2, and the EM data processing strategies are shown in *SI Appendix*, Figs. S6 and S7.

### Modeling of the FleQ^HTH^-DNA Interactions.

A model for the FleQ^HTH^ motif (residues 431 to 490) was extracted from the AlphaFold (31) structure prediction for full-length FleQ and input as search model against the PDB database in the DALI protein structure comparison server (32). The top DALI hit was for a DNA-bound FIS^HTH^ dimer and corresponded to chain A of pdb-5e3n (35) with Z-score 9, rmsd of 2 Å over all atoms and 0.57 Å over the aligned atoms, and sequence identity of 35% over the aligned residues (51/60 for FleQ and 51/91 for FIS). We thus modeled a DNA-bound FleQ^HTH^ dimer using the FIS^HTH^ dimeric assembly as a starting point and the 14-nucleotide double-stranded FleQ-binding consensus. Bond regularization on the modeled protein and DNA chains was carried out in Coot (43).

### Additional Bioinformatics tools.

Primer design was optimized using the IDT OligoAnalyzer™ tool. DNA and/or protein sequences and structures were analyzed using the ExPASy Translate and ProtParam tools (46), the AlphaFold2 structure prediction database (31), the DALI structure comparison server (32), and the PDBePISA (47) and PDBsum (48) macromolecular interface and structure exploration servers. Structure visualization was done in PyMol (Schrödinger), Chimera (49), and ChimeraX (50).

### *P. aeruginosa* Mutant Generation.

To generate Δ*fleN* and Δ*fleQ* in-frame deletion mutants in the PAO1 strain, we adapted a previously reported protocol based on the use of a suicide vector, pKNG101, which allows the selection of a double recombination event (51). Briefly, ~500-bp DNA fragments upstream and downstream of each gene were PCR amplified using PAO1 genomic DNA as a template. The fragments were then fused using overlap-extension PCR, cloned into the pCR-BluntII-TOPO vector (Invitrogen) to yield pTOPO-Δ*fleN* and pTOPO-Δ*fleQ*, sequence-verified, and subcloned using SpeI and BamHI restriction-based cloning into the pKNG101 suicide vector to yield pKNG-Δ*fleN* and pKNG-Δ*fleQ*, respectively. The suicide vectors were maintained in the *E. coli* strain CC118λpir and mobilized into *P. aeruginosa* PAO1 with the help of the *E. coli* 1,047 strain carrying the conjugative plasmid pRK2013. As the first recombination event results in the integration of the pKNG101 derivative carrying the “mutator” fragment onto the chromosome, successful recombinants were selected on *Pseudomonas* isolation agar supplemented with streptomycin at 1,000 μg/mL (52). As the pKNG101 plasmid carries the *sacB* gene, which induces cell death in the presence of sucrose, double recombinants with excised pKNG101 backbone were selected via growth on 6% sucrose-supplemented LB-agar plates and verified by PCR amplification and DNA sequencing.

To generate the in-frame PAO1 Δ*fleQ*::*fleQ*^L115D-R344E^ mutant, we amplified the wild-type *fleQ* gene together with its flanking 500-bp upstream and downstream regions and cloned it into pCR-BluntII-TOPO. Using inverse PCR, we then introduced sequentially the L^115^D and R^344^E mutations to yield pTOPO-up_*fleQ*^L115D-R344E^_down. The amplified and mutated genomic region was then subcloned in the pKNG101 suicide vector using BamHI restriction-based cloning, maintained in the *E. coli* CC118λpir strain, and mobilized via *E. coli* 1,047 pRK2013-assisted conjugation into the PAO1 Δ*fleQ* mutant. Double recombinants were selected by growth on streptomycin and sucrose as above and were verified by PCR and DNA sequencing. In addition to the in-frame *fleQ*^L115D-R344E^ mutant, the PAO1 Δ*fleQ* strain was also complemented with a wild-type copy of the *fleQ* gene as a positive control for in-frame complementation and phenotype reversal.

### Motility in Soft Agar.

Swimming motility was assessed by growth on soft agar as described previously (53). Briefly, bacteria were cultured under agitation in LB at 37 °C overnight, and then, 4 microliters of each variant were spotted on a square Petri plate containing M8 medium supplemented with 0.3% agar, 1 mM MgSO_4_, 0.5% Casamino acids, and 0.2% glucose. Plates were then incubated upright at 37 °C, and swimming was assayed after 24 h.

### Negative-Stain Electron Microscopy.

One-milliliter wild-type and mutant *P. aeruginosa* cultures were grown to exponential phase in antibiotics-free LB medium and under mild agitation to prevent surface shearing of the flagella. Cells were gently sedimented, washed with 1× tris-buffered saline, and resuspended in the same buffer. Four microliters of the cell suspension was spotted on glow-discharged carbon-coated copper grids (Agar Scientific, Stansted, United Kingdom). After ~1-min incubation, the extra liquid was blotted off, and the grids were passed sequentially through three drops of 2% w/v uranyl acetate in water, with 15 to 30 s incubation in the last drop before blotting and air-drying. Images were taken on a 200-kV Tecnai F20 Thermo Fisher transmission electron microscope equipped with an Eagle 4k × 4k CCD camera.

## Supplementary Material

Appendix 01 (PDF)

## Data Availability

All data needed to evaluate the conclusions in the paper are present in the paper and/or *SI Appendix*. Refined structural models and electron density maps have been deposited in the electron microscopy (emd-17445 (54) for the c-di-GMP-free and emd-17581 (55) for the c-di-GMP-bound FleQ-FleN complex) and protein databanks (pdb-8p53 (56) and pdb-8pb9 (57) respectively).
