# Supplementary material for: Structures of the P. aeruginosa FleQ-FleN master regulators reveal large-scale conformational switching in motility and biofilm control
Source: Proc Natl Acad Sci U S A. 2023 Dec 5;120(50):e2312276120. doi: 10.1073/pnas.2312276120 (PMC10723142; doi:10.1073/pnas.2312276120)
Supplement: Supplementary file 1 — Appendix 01 (PDF) [file pnas.2312276120.sapp.pdf]

Supplemental Material

Structures of the *P. aeruginosa* FleQ-FleN master regulators reveal large-scale conformational switching in motility and biofilm control

Lucía Torres-Sánchez[a,b,c], Thibault Géry Sana[a,b], Marion Decossas[a,b], Yaser Hashem[d] and Petya Violinova Krasteva[a,b,1]

[a] Université de Bordeaux, CNRS, Bordeaux INP, CBMN, UMR 5248, Pessac F-33600, France
[b] 'Structural Biology of Biofilms' Group, European Institute of Chemistry and Biology (IECB), Pessac F-33600, France
[c] Doctoral School of Therapeutic Innovation (ITFA). Université Paris-Saclay, Gif-sur-Yvette F-91190, France
[d] ARNA laboratory, European Institute of Chemistry and Biology (IECB), U1212 INSERM, UMR5320 CNRS, Université de Bordeaux, Pessac F-33600, France

[1] To whom correspondence may be addressed. Email: pv.krasteva@iecb.u-bordeaux.fr

This PDF includes:

Figures S1-S7
Tables S1-S2

Fig. S1: Models for bEBP-dependent transcription activation. (*A*) Model for σ^{54}-dependent transcription activation by the canonical bEBP PspF. Reported by Ye *et al.* (14) and reproduced under the CCBY 4.0 licence (https://creativecommons.org/licenses/by-nc/4.0). (*B*) Model for FleQ-FleN-dependent transcription regulation as reported by Chanchal *et al.* (26) and reproduced under the CCBY 4.0 licence (https://creativecommons.org/licenses/by-nc/4.0). Inset, crystal structure of the $FleQ^{AAA+}$-FleN complex.

Fig. S2: FleQ domain organization and expression constructs. (*A*) FleQ domain organization and constructs tested for *in cellulo* FleQ-FleN complex formation and co-purification. Right, AlphaFold prediction for full-length FleQ. The N-terminal REC domain (in green) is followed by a bilobal AAA+ domain (subdomains SD1 in deep red and SD2 in orange), an AAA+ to HTH linker partially folded in an α-helix, and a C-terminal HTH domain. (*B-C*) SDS-PAGE analysis of IMAC elution fractions for various FleQ constructs when co-expressed and purified via wild-type or mutant ^{His}FleN.

Fig. S3: FleQ and FleN conservation. Multiple sequence alignments for representative FleQ (*A*) and FleN (*B*) homologs with key residues and sequence motifs annotated.

Fig. S4: $FleQ^{HTH}$-DNA binding model. (*A*) Crystal structure of DNA-bound Fis from *E. coli*, the closest structural homolog based on AlphaFold and Dali searches. (*B-C*) $FleQ^{HTH}$ binding modeled onto the minimal FleQ-binding, pseudopalindromic, 14-bp consensus (*D*).

Fig. S5: Additional structural analyses. (*A*) Active site occupancy in the central c-di-GMP-coordinating FleQ copy in the c-di-GMP-bound complex, overlaid with the crystal structure of the AGS-bound AAA+ domain (11). For simplicity only the interacting FleQ and FleN subunits are shown on the left and only the FleQ copy on the right. (*B*) Active site occupancy in the peripheral c-di-GMP-coordinating FleQ copy in the c-di-GMP-bound complex, overlaid as in (*A*). (*C*) Compatibility of FleN monomer binding with FleQ 'spooned' oligomerization beyond the dimeric state.

Fig. S6: Sample preparation and cryo-EM data processing for the c-di-GMP-free FleQ-FleN complex. (*A*) Size-exclusion chromatography profile for the IMAC-purified c-di-GMP-free FleQ-FleN complex. The SDS-PAGE profile of the two main peaks is shown as an inset. As peak 1 fractions showed insufficient FleQ-FleN complex concentration and contamination with small ribosomal subunits, cryogrids for high-throughput data collection were prepared with peak 2 fractions. (*B*) A representative cryoelectron micrograph. (*C*) Representative views (2D class averages of the purified complex). (*D*) Data processing workflow.

Fig. S7: Sample preparation and cryo-EM data processing for the c-di-GMP-bound FleQ-FleN complex. (*A*) Size-exclusion chromatography profile for the IMAC-purified c-di-GMP-free FleQ-FleN complex. The SDS-PAGE profile of the main peak used for cryogrid preparation is shown as an inset. (*B*) A representative cryoelectron micrograph. (*C*) Representative views (2D class averages of the purified complex). (*D*) Data processing workflow.

Table S1. Strains and oligonucleotides

Table S2. Cryo-EM data collection and refinement statistics

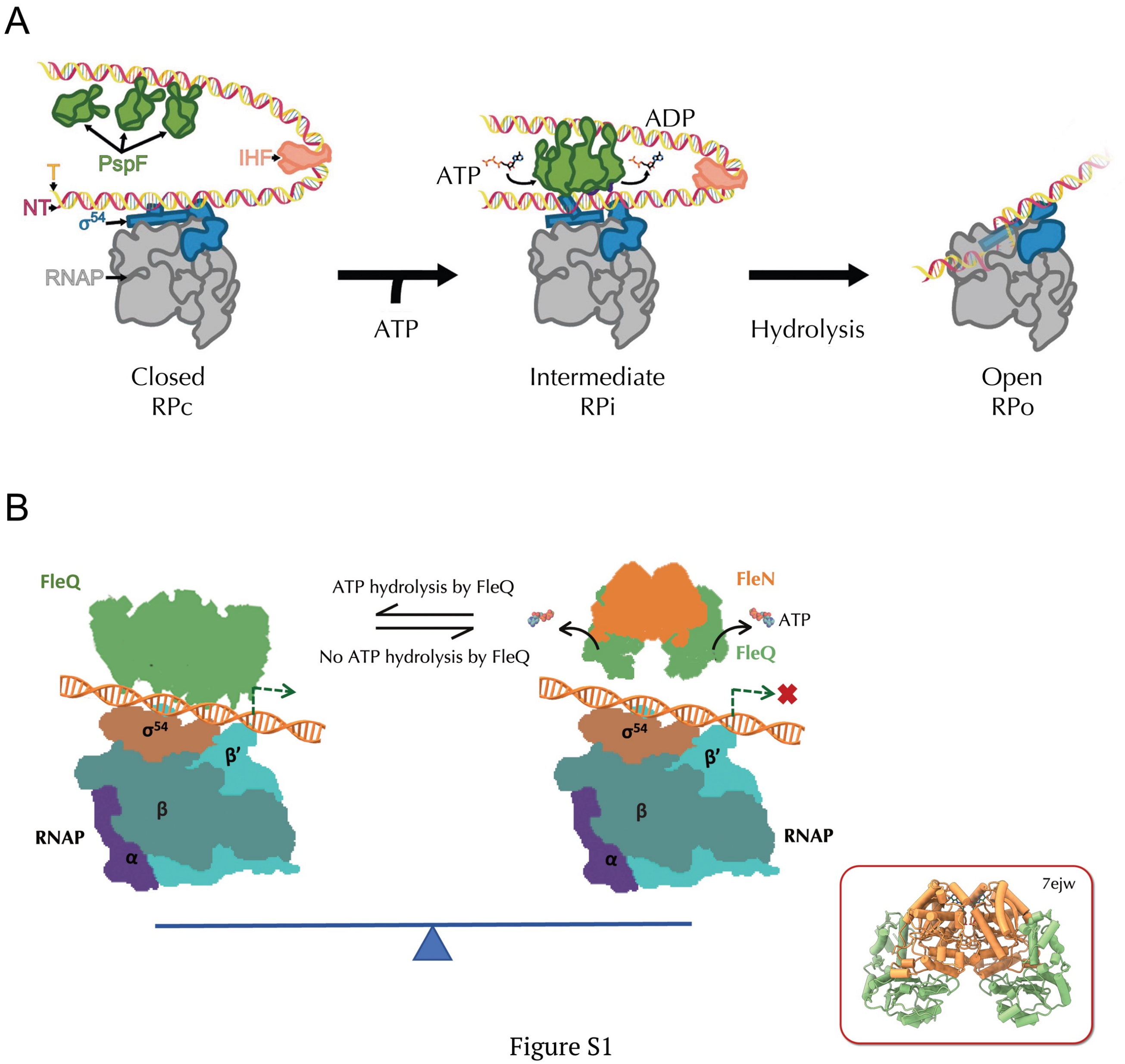
A
PspF
IHF
T
NT
σ54
RNAP
ATP
ADP
ATP
Hydrolysis
Closed
RPc
Intermediate
RPi
Open
RPo
B
FleQ
ATP hydrolysis by FleQ
No ATP hydrolysis by FleQ
FleN
ATP
FleQ
σ54
β'
β
α
RNAP
σ54
β'
β
α
RNAP
7ejw

Figure S1

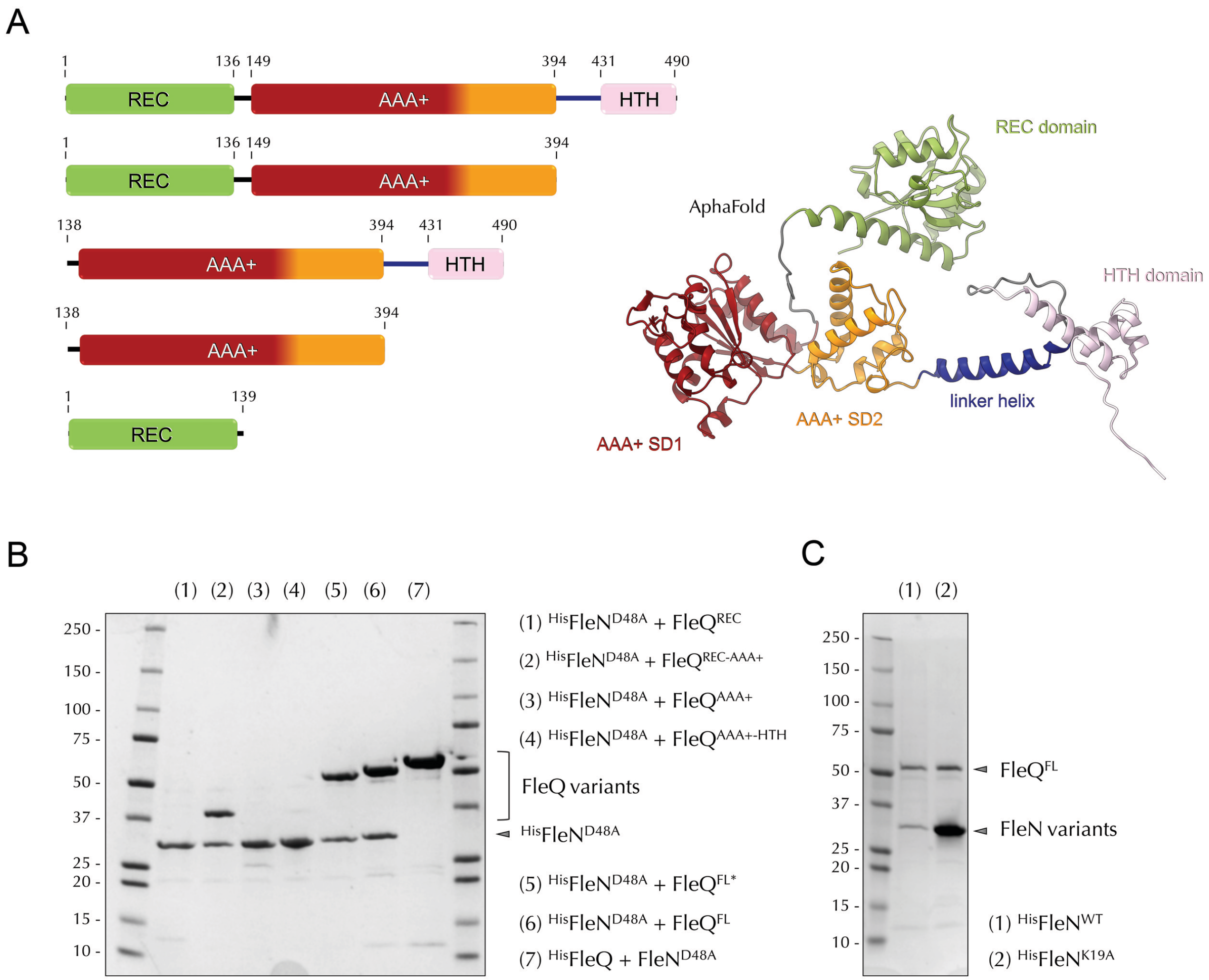

A
1
136
149
394
431
490
REC
AAA+
HTH
138
139
REC domain
AphaFold
HTH domain
AAA+ SD1
AAA+ SD2
linker helix
B
(1) (2) (3) (4) (5) (6) (7)
250 -
150 -
100 -
75 -
50 -
37 -
25 -
20 -
15 -
10 -
(1) HisFleND48A + FleQREC
(2) HisFleND48A + FleQREC-AAA+
(3) HisFleND48A + FleQAAA+
(4) HisFleND48A + FleQAAA+-HTH
FleQ variants
HisFleND48A
(5) HisFleND48A + FleQFL*
(6) HisFleND48A + FleQFL
(7) HisFleQ + FleND48A
C
(1) (2)
FleQFL
FleN variants
(1) HisFleNWT
(2) HisFleNK19A

Figure S2

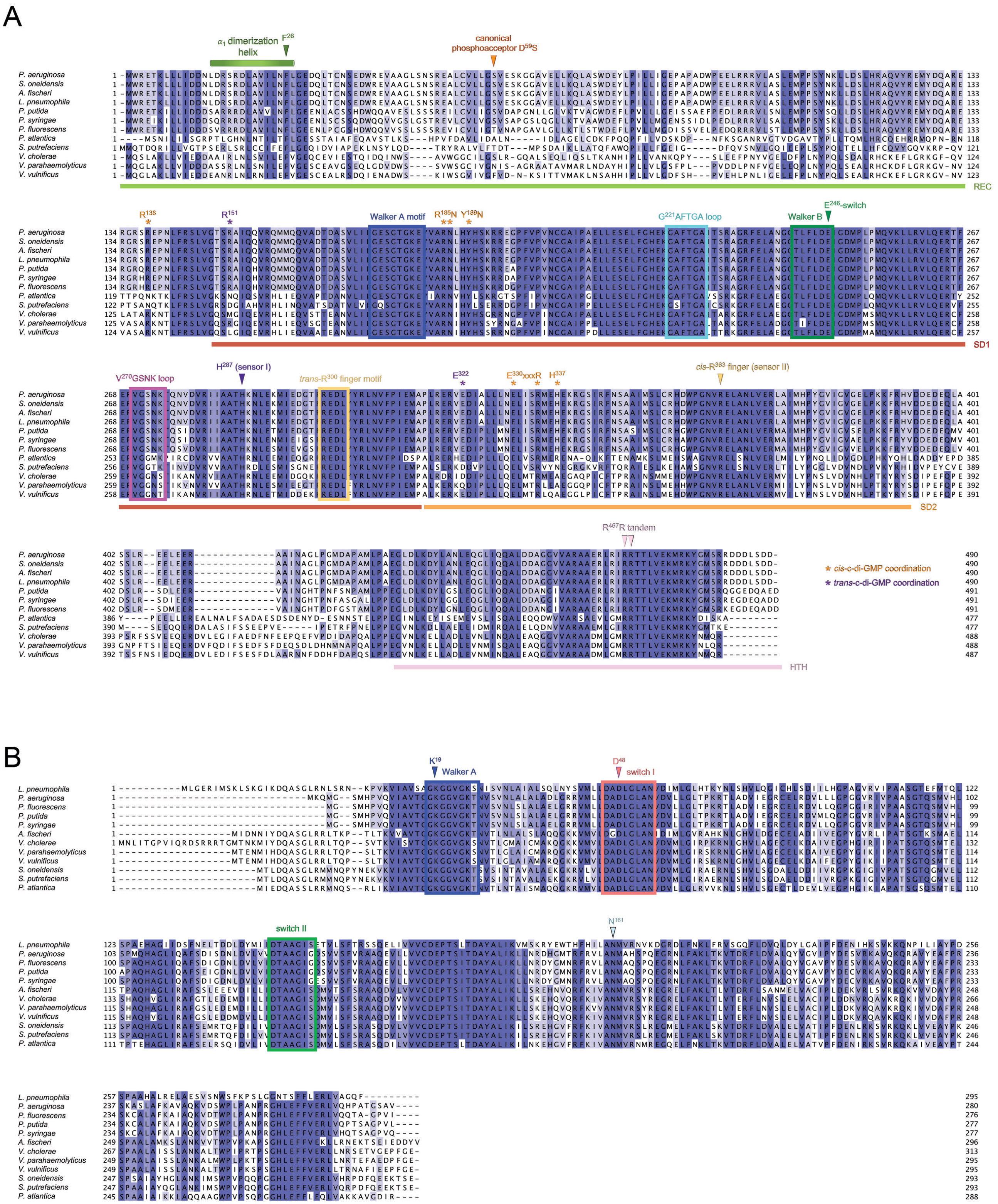
A
α1 dimerization helix
F26
canonical phosphoacceptor D59S
P. aeruginosa
S. oneidensis
A. fischeri
L. pneumophila
P. putida
P. syringae
P. fluorescens
P. atlantica
S. putrefaciens
V. cholerae
V. parahaemolyticus
V. vulnificus
REC
R138
R151
Walker A motif
R185N
Y189N
G221AFTGA loop
Walker B
E246-switch
SD1
V270GSNK loop
H287 (sensor I)
trans-R300 finger motif
E322
E330xxxR
H337
cis-R383 finger (sensor II)
SD2
R487R tandem
cis-c-di-GMP coordination
trans-c-di-GMP coordination
HTH
B
K19
Walker A
D48
switch I
switch II
N181

Figure S3

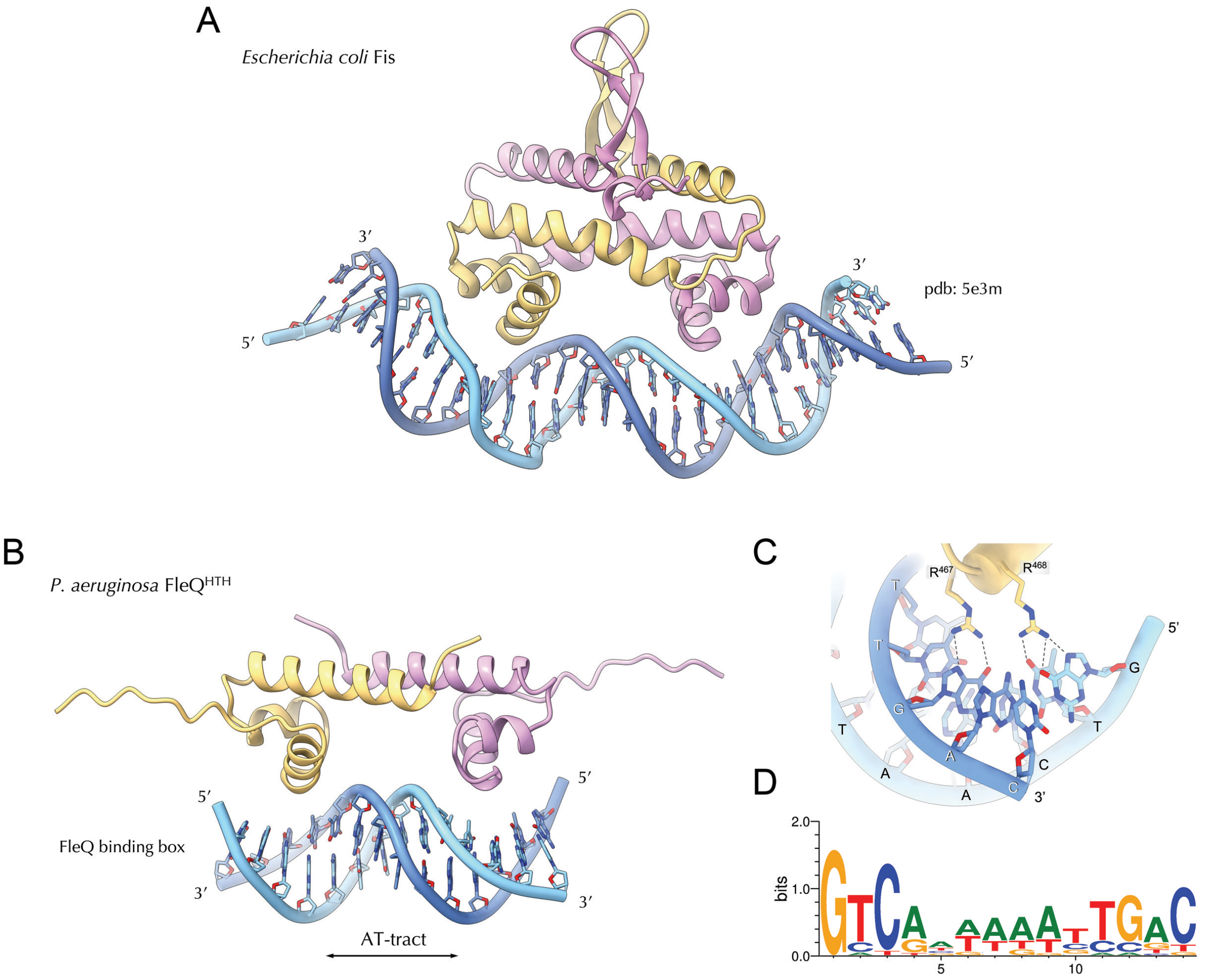
A
Escherichia coli Fis
pdb: 5e3m
3′
5′
B
P. aeruginosa FleQ^HTH
FleQ binding box
AT-tract
C
R467
R468
D
bits
2.0
1.0
0.0
5
10

Figure S4

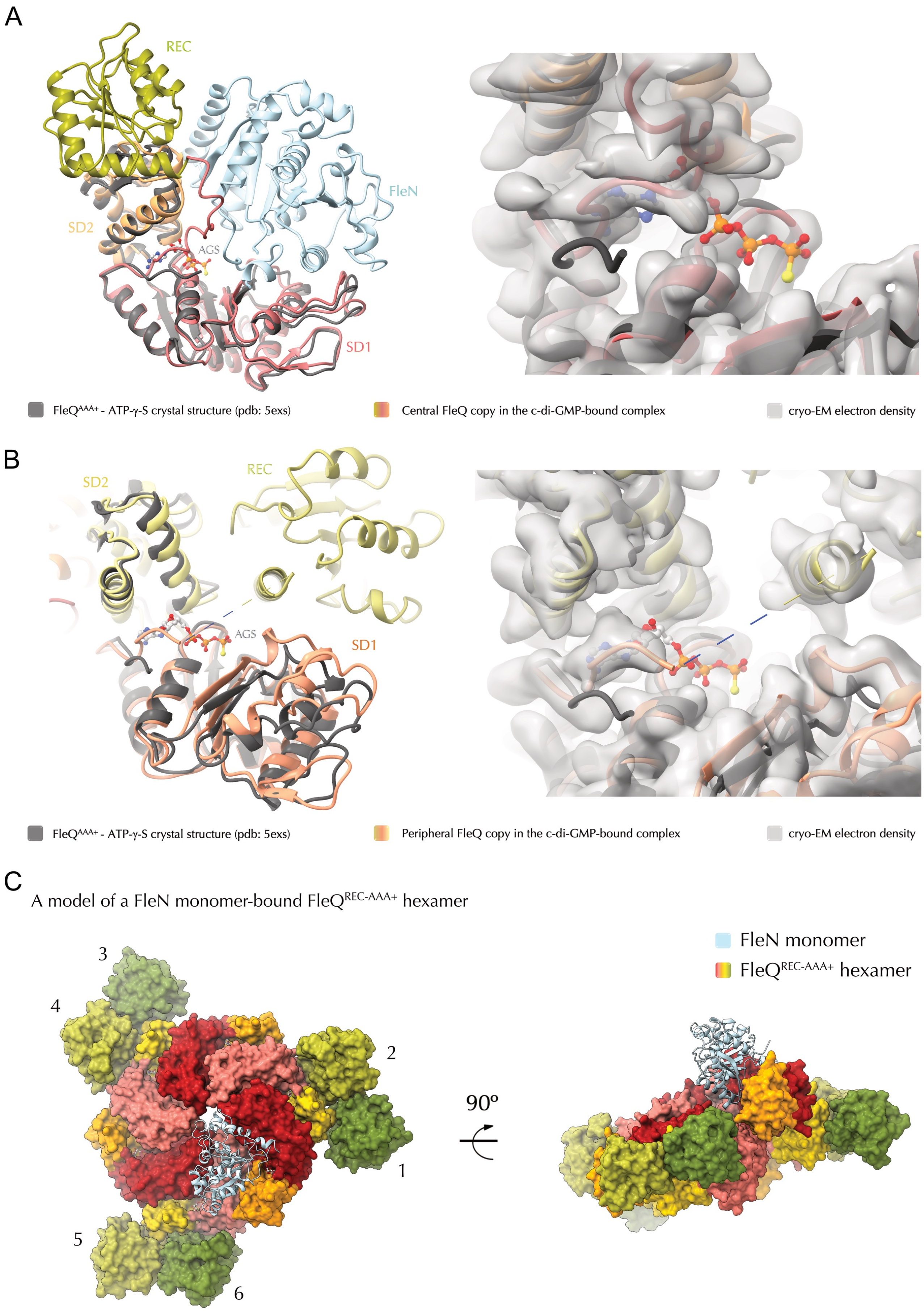
A
REC
FleN
SD2
AGS
SD1
FleQ^AAA+ - ATP-γ-S crystal structure (pdb: 5exs)
Central FleQ copy in the c-di-GMP-bound complex
cryo-EM electron density
B
SD2
REC
AGS
SD1
FleQ^AAA+ - ATP-γ-S crystal structure (pdb: 5exs)
Peripheral FleQ copy in the c-di-GMP-bound complex
cryo-EM electron density
C
A model of a FleN monomer-bound FleQ^REC-AAA+ hexamer
FleN monomer
FleQ^REC-AAA+ hexamer
3
4
2
1
5
6
90º

Figure S5

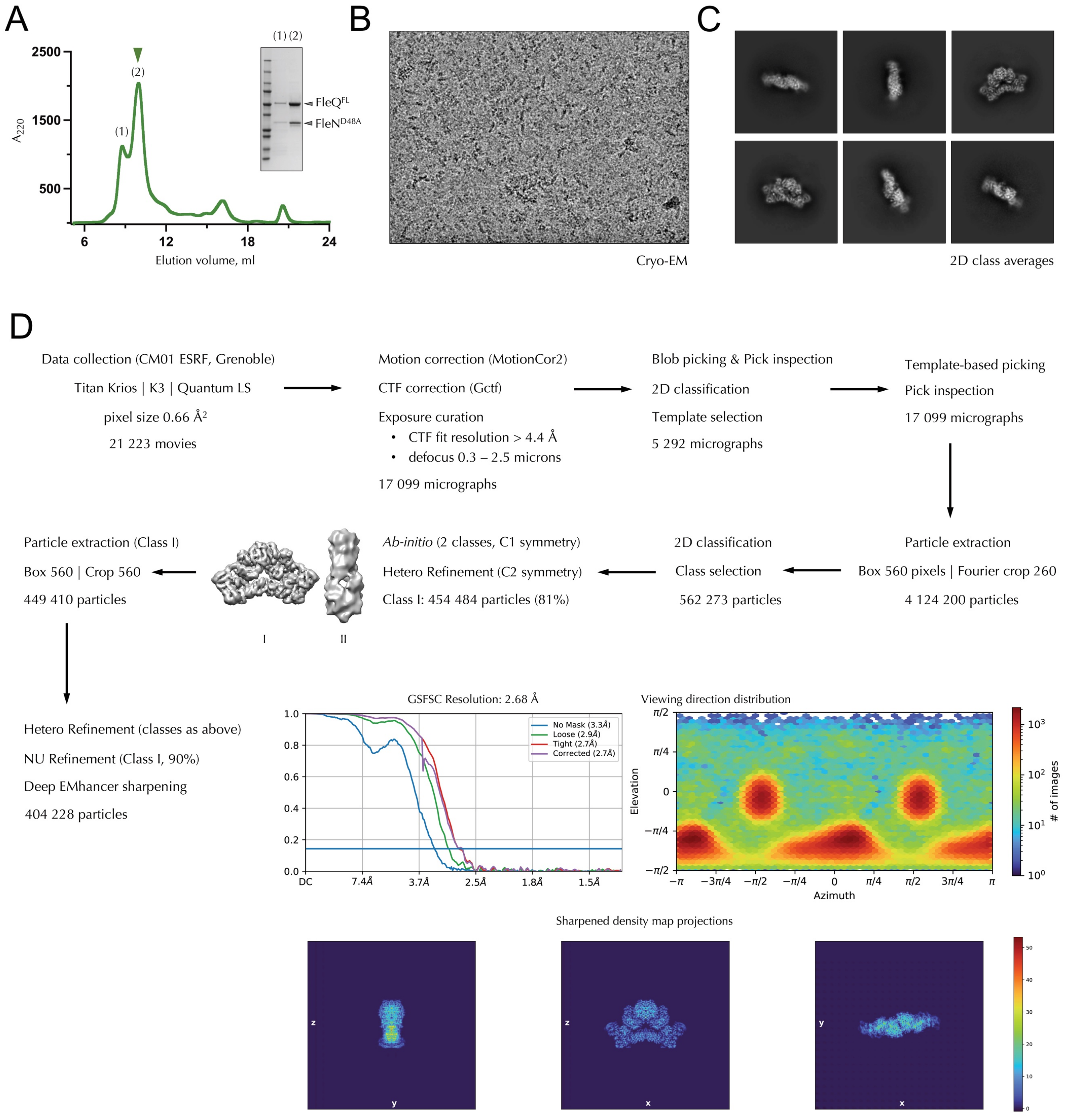
A
(1) (2)
FleQ^FL
FleN^D48A
2500
1500
500
A220
6
12
18
24
Elution volume, ml
B
Cryo-EM
C
2D class averages
D
Data collection (CM01 ESRF, Grenoble)
Titan Krios | K3 | Quantum LS
pixel size 0.66 Å²
21 223 movies
Motion correction (MotionCor2)
CTF correction (Gctf)
Exposure curation
• CTF fit resolution > 4.4 Å
• defocus 0.3 – 2.5 microns
17 099 micrographs
Blob picking & Pick inspection
2D classification
Template selection
5 292 micrographs
Template-based picking
Pick inspection
17 099 micrographs
Particle extraction
Box 560 pixels | Fourier crop 260
4 124 200 particles
2D classification
Class selection
562 273 particles
Ab-initio (2 classes, C1 symmetry)
Hetero Refinement (C2 symmetry)
Class I: 454 484 particles (81%)
I
II
Particle extraction (Class I)
Box 560 | Crop 560
449 410 particles
Hetero Refinement (classes as above)
NU Refinement (Class I, 90%)
Deep EMhancer sharpening
404 228 particles
GSFSC Resolution: 2.68 Å
No Mask (3.3Å)
Loose (2.9Å)
Tight (2.7Å)
Corrected (2.7Å)
DC
7.4Å
3.7Å
2.5Å
1.8Å
1.5Å
Viewing direction distribution
Elevation
Azimuth
# of images
Sharpened density map projections

Figure S6

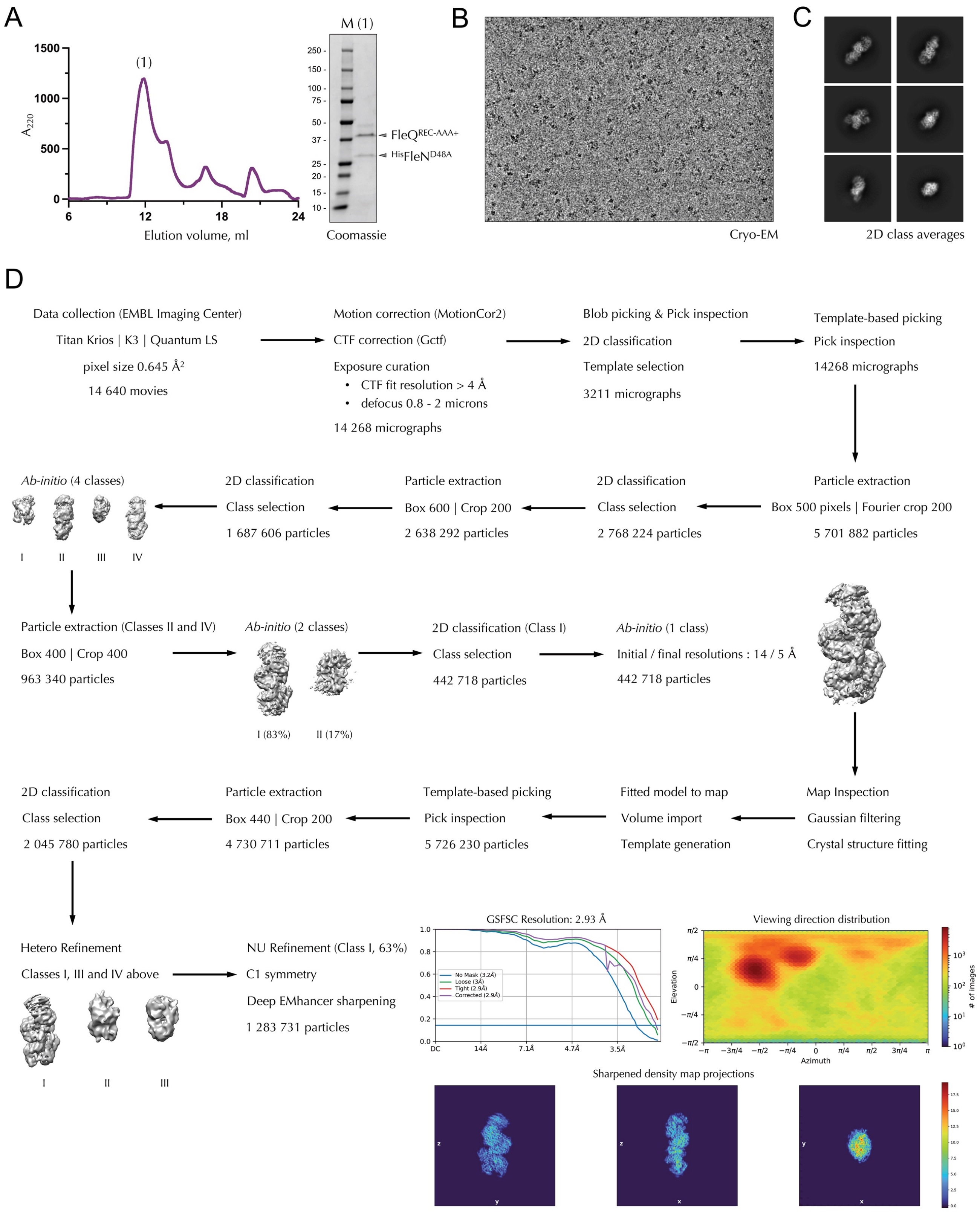

A
1500
1000
500
0
(1)
A_{220}
6
12
18
24
Elution volume, ml
M (1)
250
150
100
75
50
37
25
20
15
10
$\text{FleQ}^{\text{REC-AAA+}}$
$^{\text{His}}\text{FleN}^{\text{D48A}}$
Coomassie
B
Cryo-EM
C
2D class averages
D
Data collection (EMBL Imaging Center)
Titan Krios | K3 | Quantum LS
pixel size 0.645 Å^2
14 640 movies
Motion correction (MotionCor2)
CTF correction (Gctf)
Exposure curation
• CTF fit resolution > 4 Å
• defocus 0.8 - 2 microns
14 268 micrographs
Blob picking & Pick inspection
2D classification
Template selection
3211 micrographs
Template-based picking
Pick inspection
14268 micrographs
Particle extraction
Box 500 pixels | Fourier crop 200
5 701 882 particles
2D classification
Class selection
2 768 224 particles
Particle extraction
Box 600 | Crop 200
2 638 292 particles
2D classification
Class selection
1 687 606 particles
Ab-initio (4 classes)
I
II
III
IV
Particle extraction (Classes II and IV)
Box 400 | Crop 400
963 340 particles
Ab-initio (2 classes)
I (83%)
II (17%)
2D classification (Class I)
Class selection
442 718 particles
Ab-initio (1 class)
Initial / final resolutions : 14 / 5 Å
442 718 particles
Map Inspection
Gaussian filtering
Crystal structure fitting
Fitted model to map
Volume import
Template generation
Template-based picking
Pick inspection
5 726 230 particles
Particle extraction
Box 440 | Crop 200
4 730 711 particles
2D classification
Class selection
2 045 780 particles
Hetero Refinement
Classes I, III and IV above
I
II
III
NU Refinement (Class I, 63%)
C1 symmetry
Deep EMhancer sharpening
1 283 731 particles
GSFSC Resolution: 2.93 Å
No Mask (3.2Å)
Loose (3Å)
Tight (2.9Å)
Corrected (2.9Å)
DC
14Å
7.1Å
4.7Å
3.5Å
Viewing direction distribution
Elevation
Azimuth
# of images
Sharpened density map projections

Figure S7

Table S1 | Strains and oligonucleotides

Strain	Description and genotype	Source / Reference
Escherichia coli		
DH5α	F^- $λ^-$ *Φ80' lacZ*ΔM15 Δ(*argF-lac*) U169 *phoA supE44 recA1 relA1 endA1 thi-1 hsdR17*(r_K^-, m_K^+) *gyrA96* | Host strain for general cloning	Lab collection
BL21 Star^{TM} (DE3)	F^- *ompT hsdS*$_B$ (r_B^-, m_B^-) *GAldcmrne*131 (DE3) | Host strain for recombinant protein and protein complex expression	Lab collection
CC118λpir	Δ(*ara-leu*) *araD* Δ*lacX74 galE galK-phoA20 thi-1 rpsE rpoB argE* (Am) *recA1 Rf*^{r} (λ*pir*) | Host strain for pKNG101 replication, expresses the Pi protein for the replication of plasmids with the R6K origin	Sophie Bleves
1047 pRK2013	Kanamycin-resistant triparental conjugation helper strain. Carries the pRK2013 plasmid (Tra^+, Mob^+, ColE1, K_m^R)	Sophie Bleves
P. aeruginosa		
PAO1	Wild-type *Pseudomonas aeruginosa* PAO1 reference strain	Sophie Bleves
PAO1 Δ*fleN*	In-frame scarless deletion of *fleN* in wild-type PAO1 background	This study
PAO1 Δ*fleQ*	In-frame scarless deletion of *fleQ* in wild-type PAO1 background	This study
PAO1 Δ*fleQ*::*fleQ*$^{L115D\text{-}R334D}$	In-frame *fleQ* deletion mutant carrying a reintroduced *fleQ*$^{L115D\text{-}R344D}$ mutant gene at the endogenous chromosomal locus	This study
PAO1 Δ*fleQ*::*fleQ*^{WT}	In-frame *fleQ* deletion mutant carrying reintroduced wild-type *fleQ* gene at the endogenous chromosomal locus	This study

Oligonucleotide	Sequence	Purpose
Plasmid construction		
pRSF_MCS1_Bam_as	CATATG**GGATCC**CATGGTATATCTCCTTATTAAAG	pRSFDuet1 MCS1 redesign for BamHI/NotI-based cloning and untagged protein expression
pRSF_MCS1_Not_s	CTATAG**GCGGCCGC**ATAATGCTTAAGTCGAACAGA	pRSFDuet1 MCS1 redesign for BamHI/NotI-based cloning and untagged protein expression
Pa_FleQ_2_Bam_s	CATATG**GGATCC**TGGCGCGAAACCAAACTCTTGCTG	Used in restriction-based cloning of *fleQ*^{REC}, *fleQ*$^{REC\text{-}AAA+}$, *fleQ*^{FL}* and *fleQ*^{FL} in pRSFDuet1* and pProEx-Htb
Pa_FleQ_490_Not_as	CTATAG**GCGGCCGC**TCAATCATCCGACAGGTCGTCGTCAC	Used in restriction-based cloning of *fleQ*^{FL} *and fleQ*$^{AAA+\text{-}HTH}$ in pRSFDuet1* and pProEx-Htb
Pa_FleQ_477_Not_ as	CTATAT**GCGGCCGC**ATTACTTGCGCATCTTCTCTACCAGCG TGG	Used in restriction-based cloning of *fleQ*^{FL} in pRSFDuet1* and pProEx-Htb
Pa_FleQ_139_Not _as	CTATAT**GCGGCCGC**ATTACTCGCGGGAACGGCCGCGCTC	Used in restriction-based cloning of *fleQ*^{REC} in pRSFDuet1* and pProEx-Htb
Pa_FleQ_394_Not _as	CTATAT**GCGGCCGC**ATTAGTC GAC ATG GCG GAA TTT CTT CGG CAG	Used in restriction-based cloning of *fleQ*$^{REC\text{-}AAA+}$ and *fleQ*$^{AAA+}$ in pRSFDuet1* and pProEx-Htb
Pa_FleQ_138_Bam_s	CACATA**GGATCC**CGCGAGCCGAACCTGTTCCGCAG	Used in restriction-based cloning of *fleQ*$^{AAA+}$ and *fleQ*$^{AAA+\text{-}HTH}$ in pRSFDuet1* and pProEx-Htb
PA_FleN_2_Bam_s	CATATG**GGATCC**AAGCAGATGGGTAGCATGCATCCC	Used in restriction-based cloning of *fleN* and pRSFDuet1* and pProEx-Htb
PA_FleN_280_Not_as	CTATAG**GCGGCCGC**TCATACGGCCGAACCTGTCGC	Used in restriction-based cloning of *fleN* and pRSFDuet1* and pProEx-Htb
Point mutagenesis		
Pa_FleN_K19A_s	GGCGGCGTCGGCAAGACCAATGTGTCGGTG	Used in inverse PCR for $K^{19}A$ point mutation in *fleN*
Pa_FleN_K19A_as	CGCGCCGCCAGTTACTGCGATCACCTGTAC	Used in inverse PCR for $K^{19}A$ point mutation in *fleN*

Pa_FleN_D48A_s	GCACTCGGCCTGGCCAACGTC	Used in inverse PCR for D^{48}A point mutation in *fleN*
Pa_FleN_D48A_as	GGCATCCAGCAGCATGACGCGACG	Used in inverse PCR for D^{48}A point mutation in *fleN*
Pa_FleQ_L115D_s	ATGATTCCCTGCATCGCGCCCAGGTCTACC	Used in inverse PCR for L^{115}D point mutation in *fleQ*
Pa_FleQ_L115D_as	CCAGCTTGTTGTAGCTGGGCGGCATCTCCAG	Used in inverse PCR for L^{115}D point mutation in *fleQ*
Pa_FleQ_R344E_s	CGAATTCAACTCGGCGGCAATCATGTCGCTCTGC	Used in inverse PCR for R^{344}E point mutation in *fleQ*
Pa_FleQ_R344E_as	ATCGACCCGCGCTTCTCATGCTCCATCC	Used in inverse PCR for R^{344}E point mutation in *fleQ*
Chromosomal mutants		
fleN_500bp-up_s	TCTTCAACGTACCGGTGACC	Used to amplify a 500-bp region immediately upstream of *fleN* | Used in overlap extension PCR to fuse the up- and downstream 500-bp regions flanking *fleN*
fleN_500bp-up_as	GTTGGACCAGTCGTTCGACGACATACCTTGTGTTGTCGTCT	Used to amplify a 500-bp region immediately upstream of *fleN*
fleN_500bp-down_s	AGACGACAACACAAGGTATGTCGTCGAACGACTGGTCCAAC	Used to amplify a 500-bp region immediately downstream of *fleN*
fleN_500bp-down_as	ACTAGTCTGCAACAGGTCGTCGAAGC	Used to amplify a 500-bp region immediately downstream of *fleN* | Used in overlap extension PCR to fuse the up- and downstream 500-bp regions flanking *fleN*
fleN_700bp-up_s	CCAGGCCATGAAACTGGTCC	Used in colony PCR to verify chromosomal *fleN* gene deletion
fleN_550bp-down_as	TTCGTTGTGACTGAGGCTGG	Used in colony PCR to verify chromosomal *fleN* gene deletion
fleQ_500bp-up_s	AACTGGGATGCCATCGGTG	Used to amplify a 500-bp region immediately upstream of *fleQ* | Used in overlap extension PCR to fuse the up- and downstream 500-bp regions flanking *fleQ* | Used in amplification of the *fleQ* gene with its up- and downstream 500-bp flanking regions.
fleQ_500bp-up_as	TCAATCATCCGACAGGTCGTCCATTTTGATCAGCTGCCTTGC	Used to amplify a 500-bp region immediately upstream of *fleQ*
fleQ_500bp-down_s	GCAAGGCAGCTGATCAAAATGGACGACCTGTCGGATGATTGA	Used to amplify a 500-bp region immediately downstream of *fleQ*
fleQ_500bp-down_as	**GGATCC**ATCTCGTGACCGTCATCCTCG	Used to amplify a 500-bp region immediately downstream of *fleQ* | Used in overlap extension PCR to fuse the up- and downstream 500-bp regions flanking *fleQ* | Used in amplification of the fleQ gene with its up- and downstream 500-bp flanking regions
fleQ_650bp-up_s	AGTCAGCGCCCTGTTGTTGG	Used in colony PCR to verify chromosomal *fleQ* gene deletion
fleQ_800bp-down_as	CCTGATGCTCCAGTTCGTGC	Used in colony PCR to verify chromosomal *fleQ* gene deletion

Notes: Restriction sites are shown in bold, stop codons in red, mutation sites in purple.

Table S2 | Cryo-EM data collection and refinement statistics

Protein sample	$FleN^{D48A}{}_2$ – $FleQ^{FL}{}_4$	$FleN^{D48A}{}_2$ – $FleQ^{RE\text{-}AAA+}{}_3$
Complexed ligand	ACP/ATP_2*, $Mg^{++}{}_2$	ACP/ATP_2*, c-di-GMP_2
Data collection		
Microscope	Titan Krios (CM01 at ESRF)	Titan Krios (EMBL)
Voltage	300	300
Camera	Gatan K3	Gatan K3
Energy filter	Gatan GIF Quantum LS	Gatan GIF Quantum LS
Pixel size ($Å^2$)	0.66	0.645 (collection) / 1.419 (refinement)
Collection mode	counting with super-resolution	counting
Total electron dose ($e^-/Å^2$)	51.5	50.3
Movies	17,099	14,268
Defocus range (µm)	0.3 – 2.5	0.8 - 2
Single particles	404,228	1,283,731
Map resolution (Å)	3 Å limit for refinement	3.3 Å limit for refinement
FSC 0.143 (masked / unmasked)	2.7 / 3.3	2.9 / 3.2
Imposed symmetry	C2	none (C1)
Sharpening B-factor	112.6	135.1
Atomic model refinement		
Number of protein chains / residues	6 / 2071	5 / 1552
Number of ligands	ACP: 2 | Mg^{++}: 2	ACP: 2 | C2E: 2
Number of atoms	16,305 (hydrogens: 0)	12,266 (hydrogens: 0)
B-factors		
Protein (min/max/mean)	10.60 / 151.87 / 69.80	10.76 / 140.99 / 70.78
Ligand (min/max/mean)	26.98 / 59.31 / 47.49	22.03 / 72.64 / 45.66
Bonds (R.M.S.D.)		
Length (Å) (# > 4σ)	0.003 (0)	0.003 (0)
Angles (deg) (# > 4σ)	0.632 (4)	0.613 (0)
Ramachandran plot		
Favored (%)	96.79	95.52
Allowed (%)	3.21	4.48
Outliers (%)	0.00	0.00
Rotamer outliers (%)	0.11	0.08
Cβ outliers (%)	0.00	0.00
CaBLAM outliers (%)	1.47	1.83
Peptide plane (%)		
Cis proline / general	4.3 / 0.0	1.5 / 0.0
Twisted proline / general	0.0 / 0.0	0.0 / 0.0
CC (mask)	0.80	0.69
Occupancy		
Mean	1.00	1.00
occ = 1 (%)	100.00	99.95
0 < occ < 1 (%)	0.00	0.00
occ > 1 (%)	0.00	0.00
Clash score	8.17	13.98
MolProbity score	1.64	1.96
